# Identification of two p53 isoforms from *Litopenaeus vannamei* and their interaction with NF-κB to induce distinct immune response

**DOI:** 10.1038/srep45821

**Published:** 2017-03-31

**Authors:** Haoyang Li, Sheng Wang, Yonggui Chen, Kai Lǚ, Bin Yin, Sedong Li, Jianguo He, Chaozheng Li

**Affiliations:** 1 MOE Key Laboratory of Aquatic.Product Safety/State Key Laboratory for Biocontrol, School of Life Sciences, Sun Yat-sen University, Guangzhou, P. R. China; 2 Institute of Aquatic Economic Animals and Guangdong Province Key Laboratory for Aquatic Economic Animals, Sun Yat-sen University, Guangzhou, P. R. China; 3 Guangdong Provincial Key Laboratory of Marine Resources and Coastal Engineering, Guangzhou, P. R. China; 4 School of Marine Sciences, Sun Yat-sen University, Guangzhou, P. R. China; 5 South China Sea Resource Exploitation and Protection Collaborative Innovation Center (SCS-REPIC), Guangzhou, P. R. China; 6 Fisheries Research Institute of Zhanjiang, Zhanjiang, P. R. China

## Abstract

p53 is a transcription factor with capability of regulating diverse NF-κB dependent biological progresses such as inflammation and host defense, but the actual mechanism remains unrevealed. Herein, we firstly identified two novel alternatively spliced isoforms of p53 from *Litopenaeus vannamei* (LvΔNp53 and the full-length of p53, LvFLp53). We then established that the two p53 isoforms exerted opposite effects on regulating NF-κB induced antimicrobial peptides (AMPs) and white spot syndrome virus (WSSV) immediate-early (IE) genes expression, suggesting there could be a crosstalk between p53 and NF-κB pathways. Of note, both of the two p53 isoforms could interact directly with LvDorsal, a shrimp homolog of NF-κB. In addition, the activation of NF-κB mediated by LvDorsal was provoked by LvΔNp53 but suppressed by LvFLp53, and the increased NF-κB activity conferred by LvΔNp53 can be attenuated by LvFLp53. Furthermore, silencing of LvFLp53 in shrimp caused higher mortalities and virus loads under WSSV infection, whereas LvΔNp53-knockdown shrimps exhibited an opposed RNAi phenotype. Taken together, these findings present here provided some novel insight into different roles of shrimp p53 isoforms in immune response, and some information for us to understand the regulatory crosstalk between p53 pathway and NF-κB pathway in invertebrates.

Mammalian p53 family proteins are comprised of p53, p63 and p73, all of which have a dual gene structure with an internal promoter and can express several different isoforms through alternative splicing, alternative promoter usage, and alternative initiation sites of translation^1^. In detail, p53 gene contains 11 exons with an internal promoter in intron-4 and encodes nine different p53 protein isoforms. p63 contains 15 exons with an internal promoter in intron-3 and codes for 6 different p63 protein isoforms. The gene structure of p73 is more complex than those of p53 and p63, and p73 contains 14 exons with an internal promoter in intron-3 that expresses at least 35 mRNA variants theoretically encoding 28 different p73 protein isoforms^2^. Although a wide variety of isoforms exist in p53 family, each p53 family member executes its own unique functions in cellular progresses^3^. In regard of p53 isoforms, the functions of FLp53 and Δ133p53 isoforms, transcribed from a distal and an internal promoter located in intron-4 respectively, are well characterized, and they are shown to play an important role in many aspects such as pathogenic infection^4^.

The mammalian p53 was firstly discovered as a cellular partner of the oncogenic T antigen from SV40 virus^5^,^6^. In the last twenty years, p53 has been identified as a pivotal tumor suppressor with key roles in modulating the expression of a variety of genes involved in cell cycle arrest^7^, DNA repair^8^ and apoptosis^9^. The tumor suppressor p53 can function as a transcription factor not only to activate gene transcription, but also to repress the expression of responsive genes. Commonly, p53 can bind directly and specifically as a tetramer to target sequences of DNA through p53-responsive elements (p53REs) in proximity promoters of regulated genes^10^,^11^. Of note, p53 can regulate the expression of genes without p53REs in their promoter, by interactions with other transcriptional regulators such as NF-κB^12^. p53 was also shown to interact with p300, a general transcriptional coactivator and therefore can alter other p300-dependent factors transcriptional activity^13^,^14^. Indeed, p53 has been reported to cross talk with other signaling pathways such as NF-κB pathway and regulates diverse NF-κB dependent cellular responses^12^. Therefore, in addition to its tumor suppress function, p53 is also involved in some biological progresses in content of inflammation^15^, immune activation^16^ and host defense^17^.

Emerging evidence shows that p53 plays a complex role in viral infection^18^. The activation of p53 induced by virus can trigger host cellular sensors that initiate cell death, which plays an important protective role in eliminating viral infected cells^18^. Moreover, p53 can promote the host defense through modulating several pathways such as interferon-α/β pathways^19^ and NF-κB pathway^20^. Thus, it is generally accepted that p53 is a host restriction factor in a plethora of viral infections. For example, knockout or knockdown of p53 results in higher viral loads (or enhanced viral replication) during several viral infection including hepatitis C virus (HCV)^21^, vesiclular stomatitis virus (VSV)^22^, poliovirus^23^, influenza A virus (IAV)^24^ and JC virus^25^. On the other hand, some viruses such as human cytomegalovirus (HCMV)^26^ and herpes simplex virus 1 (HSV-1)^27^ seem to require p53 for efficient viral replication. For example, p53 knockout of mice shows lower viral replication in brains and reduces mortality in response to herpes simplex virus 1 (HSV-1) infection, suggesting that p53 may be involved in the facilitation of HSV-1 infection^28^. Besides, p53 and p53 isoforms were also shown to participate in bacterial infection. For example, pathogenic bacterium *Helicobacter pylori* up-regulates the expression of truncated p53 isoforms (Δ133p53, Δ160p53 and Δ153p53), which then inhibit p53 and p73 mediated proapoptotic activities, induce NF-κB, and thus increase survival of *H. pylori* infected cells^4^. Overall, viral and bacterial infection is a typical stress, which can activate p53 and p53 mediated cellular responses that result in different biological effects depended on the interplay between host p53 and specific stress stimuli.

To date, the mechanism regulating the activity of NF-κB pathway via p53 remains elusive. In this study, a novel alternatively spliced isoform of p53 from *L. vannamei* was identified (LvΔNp53). Both LvFLp53 (full-length of p53) and LvΔNp53 could interact with LvDorsal, a shrimp homolog NF-κB, and showed opposite functions in regulating the expression of NF-κB targeted genes. In addition, we presented evidence that the cross transcriptional interference between Lvp53 and LvDorsal was physiologically important in host defense against pathogen invasion, indicating a complex regulatory mechanism for signal transduction in the innate immunity of arthropods.

## Results

### Sequence analysis and phylogenetic tree of Lvp53

**Figure 1:**
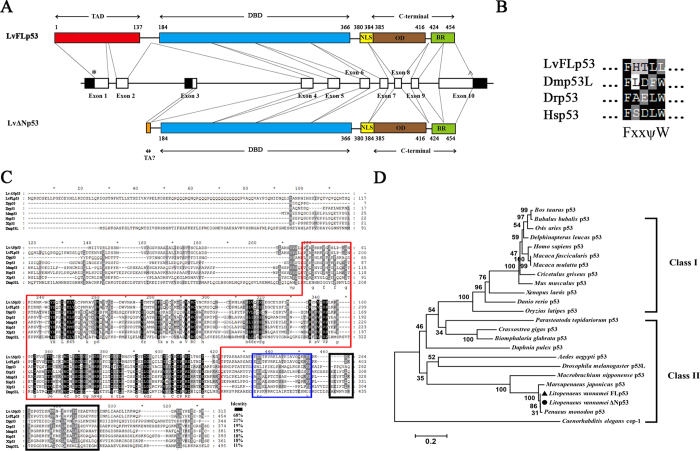
The sequence analysis of LvFLp53 and of LvΔNp53. (**A**) Scematic representation of genomic structure of p53 from *Litopenaeus vannamei.* (TAD) transactivation domain; (DBD) p53 family DNA binding domain; (NLS) nuclear localization signal; (OD) oligomerization domain; (BR) basic region. Exons (numbered boxes) were shown on the genomic sequence (horizontal line) with start and stop codons indicated by asterisks (*) and carets (^), respectively. Numbers above the bar indicated the amino acid start points of each putative domain. (**B**) Alignment of transctivation domain FxxψW corresponding to residues 32 to 36 of *L. vannamei* FLp53 (LvFLp53), residues 35 to 39 of *Drosophila melanogaster* p53 (Dmp53L), residues 9 to 13 of *Danio rerio* p53 (Drp53) and residues 19 to 23 of *Homo sapiens* p53 (Hsp53). (**C**) Multiple sequence alignment of p53 homologs. Amino acids identities of the LvΔNp53 with LvFLp53 and p53 proteins from other species were shown on the right. The conserved p53 family DNA binding domain (DBD), nuclear localization signal (NLS) and oligomerization domain (OD) were boxed with red line, blue line and black line, respectively. Proteins analyzed listed below: LvFLp53, *L. vannamei* FLp53 (KX827273); LvΔNp53, *L. vannamei* ΔNp53 (KX827274); Dpp53, *Daphnia pulex* p53 (EFX89004.1); Drp53, *Danio rerio* p53 (NP_001258749.1); Mmp53, *Mus musculus* p53 (AAA39883.1); Hsp53, *Homo sapiens* p53 (BAC16799.1); Xlp53, *Xenopus laevis* p53 (CAA54672.1); Dmp53, *Drosophila melanogaster* p53 (NP_996267.1). (**D**) Phylogenetic tree analysis of p53 proteins. Phylogenetic tree analysis were based on the full-length amino acid sequences of p53 proteins (LvFLp53 was marked with a triangle and LvΔNp53 is marked with a circle) using MEGA 5.0 software. Proteins analyzed listed below: *Bos taurus* p53 (CAA57348.1); *Ovis aries* p53 (CAA57349.1); *Bubalus bubalis* p53 (AEG21062.2); *Delphinapterus leucas* p53 (AAL83290.1); Hsp53 (BAC16799.1); *Macaca fascicularis* p53 (AAB91535.1); *Macaca mulatta* p53 (AAB91534.1); *Cricetulus griseus* p53 (AAC53040.1); Mmp53 (AAA39883.1); Xlp53 (CAA54672.1); Drp53 (NP_001258749.1); *Oryzias latipes* p53 (AAC60146.1); *Parasteatoda tepidariorum* p53 (XP_015904437.1); *Crassostrea gigas* p53 (CAJ85664.2); *Biomphalaria glabrata* p53 (XP_013061527.1); Dpp53 (EFX89004.1); *Aedes aegypti* p53 (EAT40700.1); Dmp53L (NP_996267.1); *Macrobrachium nipponense* p53 (AMW91024.1); *Marsupenaeus japonicus* p53 (BAL15075.1); LvFLp53 (KX827273); LvΔNp53 (KX827274); *Penaeus monodon* p53 (AMQ13578.1); *Caenorhabditis elegans* cep-1 (AAL28139.1).

*L. vananmei* p53 gene contained 10 exons and encoded two p53 protein isoforms, named as LvFLp53 and LvΔNp53, respectively (Fig. 1A and Supplementary Fig. 6). Of particular note, exon-3 contained an internal promoter that can be alternatively spliced to express LvΔNp53 with a unique 6-aa sequence and deletion of a 137 amino acids transactivation domain (TAD) in the N-terminal compared to LvFLp53 (full length of p53) (Fig. 1A). The genome organization and protein domains location showed the two Lvp53 isoforms shared several the same characteristic domains including p53 family DNA-binding domain (DBD), nuclear localization signal (NLS), oligomerization domain (OD) and the C-terminal basic region (BR) (Fig. 1A). Besides, we observed that LvFLp53 contained an imperfect FxxψW motif in the amino-terminal transactivation domain, while LvΔNp53 lacked this motif due to alternative splicing of intron 2 (Fig. 1B). The conservation of the transactivation domain FxxψW defined the LvFLp53 as homologous to full-length p53 in other species. Overall, *L. vannamei* p53 gene contained an internal promoter in exon-3, confirming that the *L. vannamei* p53 has a dual gene structure that is similar to p53 genes in other species. Taken together, we, in view of the above-mentioned characteristics, supposed that LvΔNp53 could be homologous to *Drosophila melanogaster* Δ124p53 (AAF56087.2), *Danio rerio* Δ113p53 (AJD19812.1) and *Homo sapiens* Δ133p53 (NP_001119587.1).

The transcript of LvFLp53 was 2210 bp in length with a 122 bp 5′-untranslated region (UTR), a 723 bp 3′-UTR containing a poly (A) tail, and a 1365 bp open reading frame (ORF) coding for a 454 amino acids protein with molecular weight (MW) of ~51 kDa and theoretical isoelectric point (pI) of 5.74 (Supplement Fig. 1A) (GenBank accession No. KX827273). LvFLp53 cDNA sequence was identical to the previous reported p53 from *L. vannamei* (KC422442.1 or KX179650)^29^,^30^ with a little discrepancy in the coding region, which may be generated by synonymous or nonsynonymous substitution. The full length of LvΔNp53 cDNA (KX827274) was 1818 bp in size containing an ORF of 948 bp that encoded a polypeptide of 315 amino acids with MW of ~34.8 kDa and theoretical pI of ~8.33 (Supplement Fig. 1B).

Multiple sequence alignment indicated that LvFLp53 and LvΔNp53 proteins shared low sequence similarities with p53 from other species ranged from 11% to 21%, but showed high homologies in the DBD, NLS and OD domain (Fig. 1C). The full-length deduced amino acid sequence of LvΔNp53 showed 68% identity to LvFLp53, 21% identity to *Daphnia pulex* p53 protein (Dpp53), 19% identity to *Danio rerio* p53 protein (Drp53), 19% identity to *Mus musculus* p53 protein (Mmp53), 18% identity to *Homo sapiens* p53 protein (Hsp53), 18% identity to *Xenopus laevis* p53 protein (Xlp53) and 11% identity to *Drosophila melanogaster* p53L protein (Dmp53L), respectively (Fig. 1C). Besides, the neighbor-joining (NJ) phylogenetic tree demonstrated that these p53 proteins could be divided into two classes, the vertebrate p53 proteins and the invertebrate p53 proteins respectively (Fig. 1D). LvFLp53 and LvΔNp53 were clustered together and shared the same branch with other arthropods in invertebrate clade (Fig. 1D), suggesting that the two *L. vananmei* p53 isoforms are the new members of p53 family.

### Tissue distribution, subcellular location and expression profile of LvFLp53 and LvΔNp53

**Figure 2:**
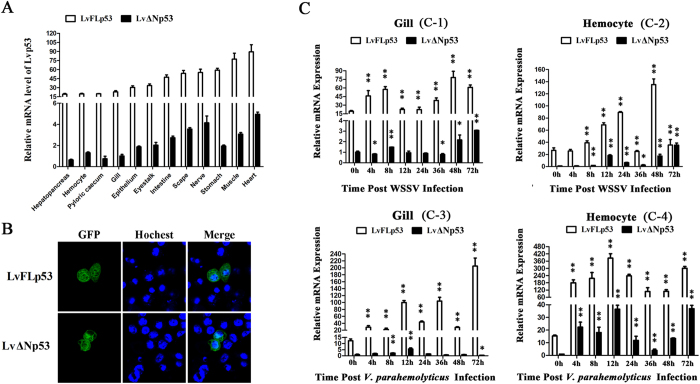
Characterization of p53 isoforms in *L. vannamei* tissues and S2 cells. (**A**) Transcription levels of LvFLp53 and LvΔNp53 in different tissues were analyzed by real-time RT-PCR. *L. vannamei* EF-1α was used as an internal control and the data were shown as mean ± SD of triplicate assays. Expression level of LvΔNp53 in the hepatopancreas was used as control and set to 1.0. (**B**) Subcellular location of LvFLp53 and LvΔNp53 in Drosophila S2 cells. S2 were transfected with plasmids pAc-LvFLp53-GFP and pAc-LvΔNp53-GFP. At 36 h post-transfection, the cells were observed using a Leica laser scanning confocal microscope. (**C**) Expression profiles of LvFLp53 and LvΔNp53 in gills or hemocytes from WSSV challenged or *V. parahaemolyticus* challenged shrimps. Real-time RT-PCR was performed in triplicate for each sample. Expression values were normalized to those of EF-1α using the Livak (2^−ΔΔCT^) method and the data were provided as the means ± SD of triplicate assays. Expression level of LvΔNp53 detected at 0 h was set as 1.0. The statistical significance between Lvp53 isoforms expression level of experiment group and that of control group treated with PBS (not shown here) was calculated using Student’s t-test (**p < 0.01 and *p < 0.05).

Both LvFLp53 and LvΔNp53 mRNA could be detected in all the examined tissues of naïve (uninfected) shrimp by real-time RT-PCR (Fig. 2A). Overall, the expression levels of LvFLp53 in all tested tissues were much higher than that of LvΔNp53 with the folds ranged from 13.3 (nerve) to 30.3 (hepatopancreas) (Fig. 2A). In detail, the expression of LvFLp53 showed high levels in heart and muscle, moderate in stomach, nerve, scape and intestine, and low in eyestalk, epithelium, gill, pyloric caecum, hemocyte and hepatopancreas (Fig. 2A). As for LvΔNp53, it was expressed abundantly in intestine, muscle, scape, nerve and heart, while extremely low in hepatopancreas and pyloric caecum (Fig. 2A).

We next detected the subcellular location of the two p53 isoforms over-expressed in Drosophila S2 cells using confocal laser scanning, and the results showed GFP-tagged LvFLp53 and GFP-tagged LvΔNp53 protein were dispersedly presented in both cytoplasm and nucleus (Fig. 2B), suggesting the location diversity of the two p53 isoforms could be independent on the variation of the N-terminal.

**Figure 3:**
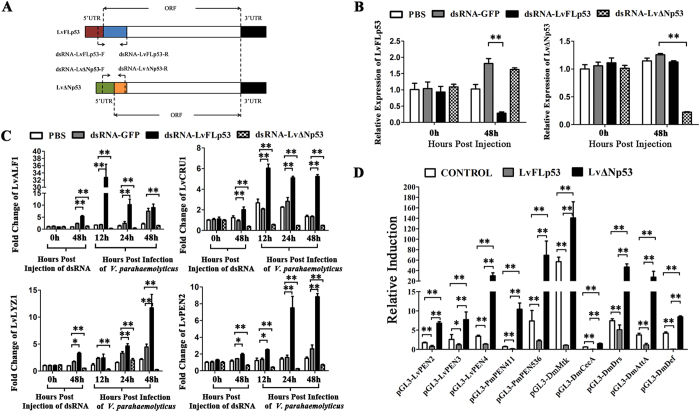
The effects of LvFLp53 and LvΔNp53 on the expression of AMPs *in vivo* and *in vitro*. (**A**) The diagram illustration of the target sequences of dsRNA-LvFLp53 and dsRNA-LvΔNp53. The primers used for the synthesis of dsRNAs were shown. (**B**) Real-time RT-PCR analysis of the silencing efficiency of LvFLp53 and of LvΔNp53. Samples were taken at 48 h after injection with the specific dsRNA or PBS. (**C**) The expression of AMPs in LvFLp53 or LvΔNp53 knockdown shrimps prior to and after *V. parahaemolyticus* infection. Shrimps were injected intramuscularly with PBS, dsRNA-LvFLp53, dsRNA-LvΔNp53 or dsRNA-GFP. At 48 h after the initial injection, shrimps were injected with *V. parahaemolyticus* or PBS as the negative control. Samples were taken at 0 and 48 h, and at 12, 24 and 48 h prior to and after *V. parahaemolyticus* infection, respectively. Bars indicated the mean ± SD of three samples and statistical significances were calculated by the Student’s t-test (**p < 0.01 and *p < 0.05). (**D**) The effects of LvFLp53 and LvΔNp53 on the promoters activities of AMPs in Drosophila S2 cells. S2 were co-transfected with the protein expression plasmids (pAc5.1-LvFLp53, pAc5.1-LvΔNp53 or the pAc5.1A-GFP as a control), the reporter gene plasmids (AMPs promoters), and the pRL-TK Renilla luciferase plasmid (as an internal control). After 48 h, the cells were harvested for measurement of luciferase activity using the dual-luciferase reporter assay system (Promega, USA). The bars indicated the mean ± SD of the luciferase activity (*n* = 6). The statistical significance was calculated using Student’s t-test (**p < 0.01).

**Figure 4:**
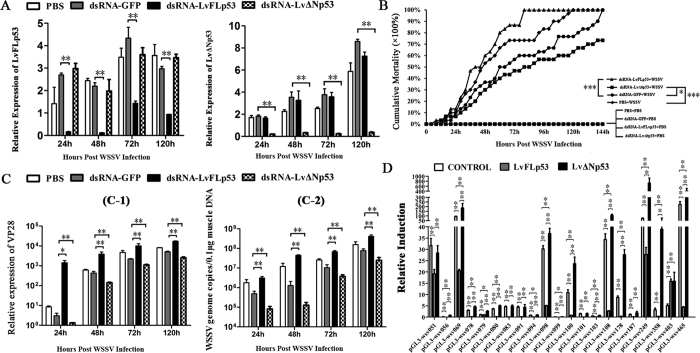
The opposing roles of LvFLp53 and LvΔNp53 during WSSV infection. (**A**) Real-time RT-PCR analysis of the silencing efficiencies of LvFLp53 and LvΔNp53. Samples were taken at 24, 48, 72, 96 and 120 h after WSSV infection from the groups with the treatment of PBS, dsRNA-LvFLp53, dsRNA-LvΔNp53 or dsRNA-GFP. (**B**) Cumulative mortality of different groups after WSSV or PBS treatment. Shrimps were injected with PBS, dsRNA-LvFLp53, dsRNA- LvΔNp53 or dsRNA-GFP. At 48 h after the initial injection, shrimps were treated with WSSV or PBS and the mortality was recorded every 4 h. (**C**) The WSSV VP28 expression levels in hemocytes (C-1) and viral genome copies in muscle tissue (C-2) of p53 knockdown shrimps and control groups. Samples were detected at 24, 48, 72 and 120 h post infection. Each bars represented the mean ± SD of three samples. Statistically significant differences are represented with asterisks (**p < 0.01 and *p < 0.05). (**D**) The effects of LvFLp53 and LvΔNp53 on the promoters’ activities of WSSV IE genes in Drosophila S2 cells. S2 cells were co-transfected with the protein expression plasmid (pAc5.1-LvFLp53, pAc5.1-LvΔNp53 or the pAc5.1A-GFP as a control), the reporter gene plasmid (WSSV IE genes promoters), and the pRL-TK Renilla luciferase plasmid (as an internal control). After 48 h, the cells were harvested for measurement of luciferase activity using the dual-luciferase reporter assay system (Promega, USA). The bars indicatec the mean ± SD of the luciferase activity (*n* = 6). The statistical significance was calculated using Student’s t-test (**p < 0.01). All analysis are performed three times with similar results.

The transcriptional changes of LvFLp53 and LvΔNp53 after pathogens challenge were measured in two immune related tissues hemocyte and gill by real-time RT-PCR (Fig. 2C). In response to WSSV challenge, the mRNA of LvFLp53 in gills was up-regulated from 4 h and maintained high expression levels in the period of infection with two peaks at 8 h and 48 h (Fig. 2C-1). The transcript of LvΔNp53 in gills was up-regulated at 4 h and 8 h, followed by recovering to the basal levels at 12 h and 24 h, and finally up-regulated gradually again from 36 h to 72 h (Fig. 2C-1). In hemocytes after WSSV infection, LvΔNp53 showed a similar expression profile with lower degree of up-regulation to that of LvFLp53, which was up-regulated from 8 h (Fig. 2C-2). With the infection of the Gram-negative bacteria *V. parahaemolyticus*, LvFLp53 expression in gills increased remarkably at 4 h, and showed a major fluctuation during the whole stage of infection (Fig. 2C-3). Different from the expression pattern of LvFLp53, LvΔNp53 in gills was slightly up-regulated at 8 h and 12 h with no obvious change at 4 h and 24~48 h, and surprisingly marginally down-regulated at 72 h (Fig. 2C-3). In hemocytes with treatment of *V. parahaemolyticus*, LvFLp53 expression was dramatically up-regulated at 4 h (~11.8-fold), and maintained significantly high levels after infection with a peak at 12 h (~25.2-fold) (Fig. 2C-4). The expression of LvΔNp53 in hemocytes ascended at 4 h and reached the first peak at 12 h (~36.48-fold), followed by receding a little until 36 h and rising again at 72 h (Fig. 2C-4).

### The effects of LvFLp53 and LvΔNp53 on the expression of antimicrobial peptides (AMPs) *in vivo* and *in vitro*

In shrimps, antimicrobial peptides (AMPs) played a major role in defense against bacterial pathogens. To explore the function of LvFLp53 and LvΔNp53 during bacterial infection, RNAi was performed to investigate the effects of LvFLp53 and LvΔNp53 on the expression of AMPs *in vivo*. We designed and synthesized two different dsRNAs namely dsRNA-LvFLp53 and dsRNA-LvΔNp53 (Fig. 3A), which could specially suppress the expression of LvFLp53 or LvΔNp53 (84.4% and 82.2% silencing efficiencies for LvFLp53 and LvFLp53 respectively), but not affect the expression of each other (Fig. 3B). We detected the expression of several types of AMPs including one Anti-LPS factor LvALF1, one Crustin LvCRU1, one Lysozyme LvLYZ1 and one Penaeidin LvPEN2 (Supplementary Fig. 4), and found that the mRNA levels of these AMPs were notably down-regulated at 48 h post dsRNA-LvΔNp53 injection in naïve (uninfected) shrimp compared to dsRNA-GFP group (Fig. 3C). We further detected the expression of these AMPs in *V. parahaemolyticus* infected shrimps, and found that *V. parahaemolyticus* could strongly induce these AMPs expression in control groups (PBS treated group and dsRNA-GFP group), but not in dsRNA-LvΔNp53 injected groups (Fig. 3C). These results suggested that LvΔNp53 could positively regulate the expression of AMPs in both naïve or uninfected shrimps and *V. parahaemolyticus* infected shrimps. Unexpectedly, LvFLp53 played an opposing role in regulating the expression of AMPs compared to that of LvΔNp53, that is, LvFLp53 negatively regulated the expression of AMPs in both prior to infection and after infection (Fig. 3C).

Dual luciferase reporter assays were next performed to judge the effect of LvFLp53 and LvΔNp53 on the regulation of AMPs *in vitro*. We observed that the expression of multiple AMPs including Drosophila AMPs DmMtk, DmCecA, DmDrs, DmAttA and DmDef, and shrimp AMPs LvPEN2, LvPEN3, LvPEN4, PmPEN411 and PmPEN536 were inhibited by LvFLp53 but promoted by LvΔNp53 (Fig. 3D), which were in good agreement with the results *in vivo*.

### The function of LvFLp53 and LvΔNp53 during WSSV infection

RNAi was conducted to investigate the role of LvFLp53 and LvΔNp53 during WSSV infection. As shown in Fig. 4A, LvFLp53 and LvΔNp53 dsRNAs can markedly suppress the expression of LvFLp53 and LvΔNp53 starting at 24 h, respectively, and they still worked until 120 h post injection.

The cumulative mortality of LvFLp53 dsRNA group was much higher than that of the GFP dsRNA group (as a positive control) (χ^2^: 11.69, *P* = 0.0006), which indicated that knockdown of LvFLp53 rendered shrimps more susceptible to WSSV infection (Fig. 4B). Surprisingly, knockdown of LvΔNp53 caused a lower cumulative mortality compared to the GFP dsRNA group (χ^2^: 3.998, *P* = 0.0456 < 0.05) (Fig. 4B), suggesting intravital LvΔNp53 might facilitate WSSV infection. Interestingly, the cumulative mortality of GFP dsRNA group (as a positive control) was lower than that of the PBS group (as a negative control), which suggested non-special dsRNA could mount shrimp antiviral immune response (Fig. 4B).

Besides, the expression levels of VP28 (WSSV envelope protein) in hemocytes tissues and WSSV genome copies in muscle tissues was also detected. As shown in Fig. 4C, the expression levels of VP28 in LvFLp53 silenced shrimps were obviously higher than those of GFP dsRNA control group with ~471.38-fold, ~8.91-fold, ~4.50-fold and ~3.41-fold increase at 24, 48, 72 and 120 h, respectively (Fig. 4C-1). Consistent with the results of VP28 detection, the higher viral loads were observed in corresponding time points after WSSV infection (Fig. 4C-2). Expectedly, both the expression levels of VP28 and WSSV genome copies was much lower in LvΔNp53 knockdown shrimps than those of GFP dsRNA control group (Fig. 4C), which correlated well with the mortality rates observed in Fig. 4B and further confirmed LvΔNp53 might be beneficial for WSSV replication.

A growing number of studies showed that WSSV genes expression, especially immediate-early genes (IEs), was required the involvement of host transcription factors such as NF-κB. To find out the regulatory relationship between LvFLp53, LvΔNp53 and WSSV, we tested the effects of LvFLp53 and LvΔNp53 on the promoter activities of 21 WSSV immediate-early genes (IEs) by dual-luciferase reporter assays in Drosophila S2 cells. The results demonstrated that LvFLp53 was able to repress the promoters’ activity of WSSV IEs, except for wsv403 (Fig. 4D). Conversely, LvΔNp53 could up-regulate the expression of most IEs such as wsv056 (~5.16-fold), wsv079 (~2.36-fold), wsv100 (~2.18-fold), wsv108 (~3.08-fold), wsv178 (~3.15-fold), wsv249 (~9.74-fold) and wsv358 (~12.11-fold) compared with control (Fig. 4D).

Together, our results suggested that LvFLp53 could restrain the expression of WSSV IEs, and thereby played an antiviral role against WSSV infection. In contrast to LvFLp53, LvΔNp53 could promote the expression of WSSV IEs, which may contribute to WSSV infection.

### Modulating NF-κB mediated immune response by LvFLp53 and LvΔNp53

**Figure 5:**
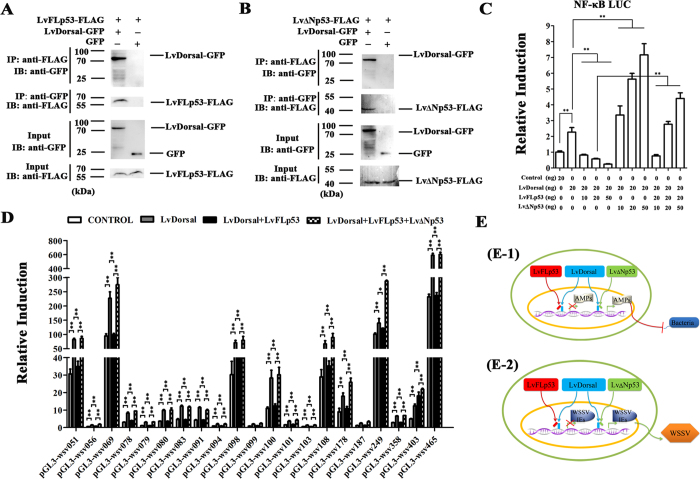
The crosstalk between shrimp p53 and NF-κB pathways. (**A**,**B**) The interaction between LvFLp53/LvΔNp53 and LvDorsal. Co-immunoprecipitation assays showed that the GFP-tagged LvDorsal but not the control GFP protein could be co-precipitated by FLAG-tagged LvFLp53 (**A**) and FLAG-tagged LvΔNp53 (**B**), respectively. Immunoprecipitation (IP) and western-blotting are performed using anti-V5 and anti-GFP antibodies, respectively. Approximate molecular sizes: LvFLp53-FLAG, ~54 kDa; LvΔNp53-FLAG, ~38 kDa; LvDorsal-GFP, ~72 kDa; GFP, ~28 kDa. (**C**) The effects of LvFLp53 and LvΔNp53 on the activity of NF-κB (LvDorsal). Drosophila S2 cells are co-transfected with pAc5.1-LvDorsal and an increasing amount of pAc5.1-LvFLp53 and/or pAc5.1-LvΔNp53, and the effects on the promoter containing four NF-κB binding domains were detected by dual luciferase reporter assays. (**D**) The effects of LvDorsal or LvDorsal co-expressed with LvFLp53 or/and LvΔNp53 on the promoters activities of WSSV IE genes. Drosophila S2 cells are co-transfected with pAc5.1-LvDorsal or/and pAc5.1-LvFLp53 and/or pAc5.1-LvΔNp53, and the effects were detected by dual luciferase reporter assays. (**E**) A possible regulatory mechanism of LvFLp53 and LvΔNp53 in NF-κB mediated immune response (See in details in discussion). All results are representative of three independent experiments.

There is a direct interaction between p53 and RelA in mammal. Besides, as observed above, both LvFLp53 and LvΔNp53 could regulate the expression of shrimp AMPs and WSSV IEs, and most of them have been proved to be regulated by shrimp NF-κB pathway^31^,^32^,^33^, indicating a potential cross talk between NF-κB and p53 signaling pathways. Interestingly, co-immunoprecipitation assay demonstrated that both LvFLp53 and LvΔNp53 could interact with LvDorsal, an NF-κB transcription factor of shrimp Toll pathway and the shrimp homolog of mammalian RelA. As shown in Fig. 5A, GFP tagged LvDorsal was co-immunoprecipitated with FLAG tagged LvFLp53 using anti-FLAG antibody, but no appreciable binding was observed for the control GFP protein. Conversely, LvFLp53 was also co-immunoprecipitated with GFP tagged LvDorsal using anti-GFP antibody. Meanwhile, the GFP tagged LvDorsal but not the control GFP can be co-immunoprecipitated with LvΔNp53-FLAG using anti-FLAG antibody, and vice versa (Fig. 5B). Accordingly, it was convenient to consider that LvFLp53 and LvΔNp53 could alter the NF-κB mediated immune response in shrimp. In previous study, an artificially modified reporter plasmid containing four repeats of NF-κB binding motifs was generated, and it can be used to detect the activation of NF-κB or corresponding pathway^34^, which was further confirmed in this assay of that LvDorsal can up-regulate the activity of NF-κB luciferase reporter indeed (Fig. 5C, lane 2). As shown in Fig. 5C, the activity of NF-κB luciferase reporter induced by LvDorsal was grossly inhibited by LvFLp53 but further promoted by LvΔNp53 in a dose dependent manner (Fig. 5C). In addition, the increased NF-κB activity conferred by LvΔNp53 was attenuated by LvFLp53 (Fig. 5C). Combined, our results showed that LvFLp53 could suppress the activity of NF-κB, but which was enhanced by LvΔNp53. To further identify that LvFLp53 and LvΔNp53 alter the NF-κB mediated immune response, we firstly established that all the 21 WSSV IEs can be positively regulated by LvDorsal manifested by dual reporter assay (Fig. 5D). When co-expressed LvDorsal with LvFLp53, the promoters activities of all WSSV IEs except wsv403 was suppressed compared to those of expressed LvDorsal alone. Interestingly, the promoters activities were repaired when co-expressed the three proteins including LvDorsal, LvFLp53 and LvΔNp53. These results shown in Fig. 5D were very similar to those of Fig. 5C. Besides, we also explored the effects of knockdown of the two p53 isoforms by RNAi *in vivo* on the expression of LvDorsal. The results demonstrated that both the transcript levels of LvDorsal mRNA in gills and hemocytes and the subcellular distribution of the LvDorsal protein in hemocytes were not changed obviously (Supplementary Fig. 2), which indicated that the variance of two p53 could mainly lead to the change of LvDorsal activity but not its transcript level. Taken together, we supposed that both LvFLp53 and LvΔNp53 could modulate NF-κB mediated immune response by targeting to the NF-κB transcription factor LvDorsal, which induced the change of LvDorsal activity and thus resulted in different immune response to bacterial and viral infection (Fig. 5E, See details in discussion).

## Discussion

It is very fascinating but poorly understood how a single protein, p53, can orchestrate diverse cellular biological processes in response to so many stress signals. A growing number of studies support the idea that p53-mediated cell response needs to be considered as isoform-specific and is, in fact, the sum of the activities of the coexpressed p53 isoforms^2^. In shrimp, p53 is reported to be implicated with host survival via its regulation roles on MnSOD and GPx in response to acute environmental stresses^29^, and p53 also plays an important role in apoptosis in response to hypoxia^30^, nevertheless, the contribution of shrimp p53 isoforms mediated immune response to pathogenic infection still remains unrevealed. In the current study, we cloned a novel alternatively spliced form of p53 from *L. vannamei* named as LvΔNp53, and then evaluated the regulatory roles of LvΔNp53 and LvFLp53 (full length of p53) on host immune genes and their function during viral infection.

Similar to p53 family in other species, the *L. vannamei* p53 gene has a dual gene structure with an internal promoter, which is highly conserved through evolution. The presence of several functional domains in LvFLp53 including a transactivation domain (TAD), a DNA-binding domain (DBD), a nuclear localization signal (NLS) and a C-terminal basic region (BR) domain suggest it may have a similar function as its counterparts in other species. Of note, LvΔNp53 transcribed from the internal promoter located in exon-3, lacks the 145 first amino acids of LvFLp53 and therefore loses the N-terminal conserved TAD, which is replaced by 6 different ones. This situation is rare in other species, except for Drosophila Δ124p53 with a unique 13 amino acids encoded by a cryptic exon replacing the N-terminal 123 ones in Drosophila FLp53^2^. Despite of lacking the TAD, LvΔNp53 is still able to transactivate the promoters of AMPs and WSSV IEs (Figs 3 and 4), and similar situation is commonly observed in p53 family that their abilities to regulate target genes are independent with the completeness of TAD, which suggests the TAD is not essential for transactivation^35^,^36^,^37^.

Knowledge of the subcellular location of proteins can provide useful insights about their biological functions such as signaling transduction and protein-protein interaction. Commonly, different subcellular localization reflects it has distinct biological activities in specific cellular environment. Although the significant differences existed in the N-terminal, LvFLp53 and LvΔNp53 were localized in both the cytoplasm and nucleus, which is consistent with the putative function of LvFLp53 and LvΔNp53 as transcription factors in capable of shuttling between the nucleus and the cytoplasm for signaling transduction and target genes expression. It is also suggested the subcellular locations of them are not related to their N-terminal amino acids, which is further supported by the observation that human full-length p53 and Δ133p53, bearing the different N-terminal, have the same subcellular location in the nucleus and in the cytoplasm^1^. Interestingly, subcellular locations of p53 isoforms are demonstrated to be associated with their carboxy-terminal amino acids^2^. For example, differing only by the last 15 carboxy-terminal amino acids, human Δ133p53β is preferably localized in the nucleus, but Δ133p53γ is exclusively localized in the cytoplasm, indicating that the C-terminal amino acids can modify the subcellular localization of these isoforms^2^.

p53 is a central sensor of cell signals and serves as a master regulator of cell response to a wide variety of stresses including pathogenic infection^17^. In this report, we find that the two p53 isoforms are expressed in a wide range of shrimp healthy tissues in a tissue-dependent manner, suggesting that the internal promoter and the splicing of p53 can be regulated. Besides, after challenged with *V. parahemolyticus* and WSSV, the elevated levels of LvFLp53 and LvΔNp53 suggest a critical role of Lvp53 in the cellular immune response to pathogenic infection. Particularly worthy of note is that the basal expression of LvFLp53 in uninfected shrimp is more abundant than that of LvΔNp53, likewise, a higher induction expression level of LvFLp53 after pathogenic infection is observed. These findings indicate the two p53 isoforms are tightly and differentially regulated in various tissues and after disparate stimuli and suggest the two p53 isoforms might execute different functions to accurately trigger appropriate cellular response to bacterial and viral infection.

As discussed above, both LvFLp53 and LvΔNp53 can be responsive to *V. parahemolyticus* infection with different expression profiles in gill and hemocyte tissues, which suggest a participation of the two isoforms in bacterial infection. We next explore the effects of the two isoforms on regulating AMPs *in vivo* and *in vitro*, which are considered as the direct effector molecules against bacteria^38^. Interestingly, we observed the opposing effects of LvFLp53 and LvΔNp53 on the expression of AMPs *in vivo* both prior to infection and post infection. Similar results were obtained with dual reporter analysis *in vitro*. Therefore, we conclude, in contrast to LvFLp53, LvΔNp53 plays a positive role in regulating the expression of AMPs and thus execute an anti-bacterial function. We further investigate the function of LvFLp53 and LvΔNp53 during WSSV infection, especially their regulatory role for viral immediate-early (IE) genes. Viral IE genes encode some regulatory proteins critical for the viral life cycle and their expression relies solely on host proteins^39^,^40^,^41^. Thus, identification of their regulatory roles for IEs could help us to understand interplay between host protein and the early stages of viral infection. Interestingly, a similar regulatory role for WSSV IE genes was observed by ectopic expression of LvFLp53 and LvΔNp53 in Drosophila S2 cells. Of note, because of LvΔNp53 positively regulating viral IE genes expression, therefore, the activation of LvΔNp53 could contribute to viral infection, which is different from bacterial infection. Altogether, our results indicated that the shrimp p53 isoforms differ in their function in response to viral and bacterial infection.

Accumulating evidence indicates reciprocal regulation of NF-κB and p53 pathways exists at multiple regulatory levels. In this study, we established by coimmunoprecipitation assay that both LvFLp53 and LvΔNp53 can form a protein complex with LvDorsal (NF-κB) homologous to human RelA, which is in line with the previous report in human of the direct interaction between these two transcription factors through their dimerization/tetramerization domains^12^. In addition, as mentioned earlier, LvFLp53 can inhibit the expression of AMPs and WSSV IEs except wsv403, both of which, of note, are demonstrated to be positively regulated by LvDorsal. In contrast to LvFLp53, LvΔNp53 plays a positively regulatory role on the expression of AMPs and WSSV IEs, suggesting LvΔNp53 could act in a dominant negative manner toward LvFp53, and thus has a similar function to NF-κB. Taken together, these findings are in good agreement with the general concept of “functional antagonism” between p53 and NF-κB pathways^20^. Furthermore, considering LvΔNp53 showing the much lower degree of expression levels in both the pre-invasion and post-invasion periods compared to LvFLp53, LvFLp53 could preferentially play a dominant role in shrimp innate immune response, but there could be a finely tuned mechanism mediated by LvΔNp53. For example, LvΔNp53 can serve as a negative regulator of LvFLp53 to avoid hyper-activating immune response that is harmful to host. Interestingly, many NF-κB binding sites instead of p53 binding motif are present in the promoter region of AMPs and WSSV IEs except wsv403. Besides, based on interaction between p53 and NF-κB, we conclude that both LvFLp53 and LvΔNp53 regulate (promote or inhibit) the expression of AMPs and WSSV IEs through their ability to bind with LvDorsal. In light of that the promoter of wsv403 contains both NF-κB binding site and p53 binding site (Supplementary Fig. 5), the regulatory outcome of wsv403 by LvFLp53 and LvΔNp53 is more complex, which could be explained why it is different from others.

In our previous studies, shrimp NF-κB (LvDorsal) pathway is shown to play a vital role in defense against invading bacteria^32^. Upon bacterial infection, the cytoplasmic NF-κB is activated rapidly and translocates into nucleus to stimulate the expression of AMPs fighting against invaders^42^. Besides, it is worth emphasizing that NF-κB pathway is also shown to be engaged by WSSV for genes transcription and genome replication^33^. Briefly, the NF-κB pathway is activated after WSSV infection, and the activated NF-κB could enhance the expression of WSSV IEs and in turn promote viral replication^33^. In this study, our findings supply a novel regulatory layer at the transcriptional crosstalk between p53 and NF-κB pathways (Fig. 5E). In response to bacterial infection, NF-κB mediated the expression of AMPs was modulated by both LvFLp53 and LvΔNp53, which could be selectively used by *L. vannamei* for producing different levels of AMPs to defense against bacterial invasion and also contribute to create a fine tuning effect to prevent hyper-immune response. However, it may be a different situation during virus infection. In addition to that LvFLp53 can suppress the expression of WSSV IEs by targeting with NF-κB (Dorsal), *L. vannamei* p53 (sequence identical to LvFLp53) has been reported to play a cytoprotective role during the basal apoptotic program in hemocytes^30^, which may be an important antiviral mechanism. As for LvΔNp53, its interaction with NF-κB could induce the expression of WSSV IEs, which is propitious to WSSV infection. In view of LvΔNp53′s lower expression levels compared to that of LvFLp53, the additive effects of LvFLp53 and LvΔNp53 on WSSV may be antiviral. Although interplay between p53 and NF-κB is complex and still unclear, intersection and crosstalk between the p53 and NF-κB pathways maybe act in a cooperation manner to determine the appropriate cellular response to pathogenic infection, and a further investigation needs to address the nature of this action.

## Materials and Methods

### Animals and pathogens

Healthy *L. vannamei* (4~6 g weight each) were purchased from the local shrimp farm in Zhanjiang, Guangdong Province, China, and cultured in a recirculating water tank system filled with air-pumped sea water with 2.5% salinity at 27 °C, and fed to satiation three times/day on a commercial diet. The Gram-negative *V. parahaemolyticus* were cultured in Luria broth (LB) medium overnight at 37 °C. Bacteria were quantified by counting the microbial colony-forming units (CFU) per milliliter on LB agar plates. The final injection concentration of *V. parahaemolyticus* should be controlled to yield ~1 × 10^5^ CFU/50 μL. WSSV was extracted from the WSSV-infected shrimp muscle tissue and stored at −80 °C. Before injection, muscle tissue was homogenized and prepared as WSSV inoculum with ~1 × 10^5^ copies in 50 μL PBS following a published method^43^.

### Cloning of full length of LvFLp53 and LvΔNp53 cDNA

An expressed sequence tag (EST) encoding a partial p53 family protein was retrieved from shrimp transcriptome data^44^ to design gene specific primers (Table 1) to obtain the 3′ and 5′ end of *L. vannamei* p53 genes using the rapid amplification cDNA ends (RACE) method. The RACE-PCR allows specific amplification of only capped mRNA, which is a common method to determine the transcription initiation sites of the target gene. The cDNA template for RACE-PCR was prepared with the SMARTer PCR cDNA Synthesis Kit (Clontech, Japan). The conditions of the RACE-PCR were the same as previous research^45^. The final PCR products were cloned into pMD-19T Cloning Vector (TaKaRa, Japan) and 12 positive clones were selected and sequenced.

### Amplification of genomic p53 genes in *L. vannamei*

The *L. vannamei* genome DNA was prepared as we mentioned in the paper previously reported^46^. The primer pairs of GLvp53-F1/GLvp53-R1, GLvp53-F2/GLvp53-R2, GLvp53-F3/GLvp53-R3, GLvp53-F4/GLvp53-R4, GLvp53-F5/GLvp53-R5 and GLvp53-F6/GLvp53-R6 (listed in Table 1 and Supplementary Fig. 6) were designed from the corresponding cDNA sequences, and were used for the genome amplification. The final genomic sequence was gained by overlapping with their adjacent fragments.

### Sequence and phylogenetic analysis of LvFLp53 and LvΔNp53

Protein domains of LvFLp53 and LvΔNp53 were identified by using Simple Modular Architecture Research Tool (SMART) (http://smart.embl.de/). Protein sequences of p53 in other species were collected from NCBI database by BLAST searches. We aligned LvFLp53, LvΔNp53 and their homologs using Clustal X v2.0 program^47^ and GeneDoc software where the identities between LvFLp53, LvΔNp53 and others were labeled. The neighbor-joining (NJ) phylogenic tree was constructed based on the deduced amino acid sequences by utilizing MEGA 5.0 software^48^.

### The real-time RT-PCR analysis of LvFLp53 and LvΔNp53 expression

The shrimp tissues including hemocyte, muscle, eyestalk, scape (the first segment of antennae), gill, epithelium, hepatopancreases, intestine, stomach, heart, nerve and pyloric ceca were sampled. Three samples from each tissue were collected from 15 shrimps (5 shrimps pooled together) for tissue distribution assay.

For pathogens challenge experiments, 200 shrimps were divided into two experimental groups (100 shrimps in each group), in which each shrimp was injected with ~1 × 10^5^ CFU of *V. parahaemolyticus* or ~1 × 10^5^ copies of WSSV particles in 50 μl PBS, respectively. The negative control group (100 shrimps) was set and received an injection of 50 μl PBS only. Gills and hemocytes of challenged shrimps were collected at 0, 4, 8, 12, 24, 36, 48, 72 h post injection, and 3 samples at each time point were pooled from 9 shrimps (3 shrimps each sample). Total RNA was isolated from each sample by utilizing the Rneasy Mini kit (QIAGEN). The first-strand cDNA synthesis was performed with PrimeScript RT Reagent Kit (Takara). Reactions of the real-time PCR were performed in the LightCycler 480 System (Roche, Germany) using SYBR Green Master Mix (Takara). All samples were tested in triplicate. Primer sequences were listed in Table 1.

### Plasmid construction

The 3 × FLAG coding sequence (Table 1) was synthesized and cloned into pAc5.1/V5-His A (Invitrogen) at BstBI/PmeI sites to replace the V5-His tag, generating a pAc5.1-FLAG vector for FLAG-tagged protein expression. The open reading frame (ORF) of LvFLp53 or LvΔNp53 without a stop codon were constructed into pAc5.1/V5-His A (Invitrogen), pAc5.1-GFP^43^ and pAc5.1-FLAG vectors at KpnI and ApaI sites for expressing V5-tagged, GFP-tagged and FLAG-tagged LvFLp53 or LvΔNp53 fusion proteins, respectively. As the same way, the vectors expressing V5-fusion and GFP-fusion LvDorsal (FJ998202) were also generated at KpnI and NotI sites^32^.

The reporter gene plasmids containing *L. vannamei* AMPs promoters including Penaeidin2 (LvPEN2), Penaeidin3 (LvPEN3) and Penaeidin4 (LvPEN4), *Penaeus monodon* AMPs promoters including Penaeidin411 (PmPEN411) and Penaeidin536 (PmPEN536), Drosophila AMPs promoters including Metchnikowin (Mtk), Cecropin A (CecA), Drosomycin (Drs), Attacin A (AttA) and Defensin (Def) were constructed using the primers showed in Supplementary Fig. 3. The reporter gene plasmids containing the 5′ flanking regions of 21 white spot syndrome virus (WSSV) immediate-early (IE) genes were also constructed with corresponding primers (Supplementary Fig. 5). The NF-κB luciferase (LUC) plasmid with an artificial promoter containing four-tandem NF-κB-binding sites was obtained from previous study^34^.

### Dual-luciferase reporter assays

Given that no permanent shrimp cell line was available, Drosophila Schneider 2 (S2) cell line was used to perform the functional analysis of LvFLp53 and of LvΔNp53. S2 cells were cultured at 28 °C in Schneider’s Insect Medium (Sigma, USA) containing 5% fetal bovine serum (Gibco, USA). For dual-luciferase reporter assays, S2 cells were plated into a 96-well plate (TPP, Switzerland). After cell reaching 60% confluence, the cells of each well were transfected with 0.05 μg firefly luciferase reporter gene plasmids containing promoter region, 0.005 μg pRL-TK renilla luciferase plasmids (Promega, USA) as internal control, and 0.02 μg (or indicated value) expression plasmids (or pAc.5.1-GFP expression plasmids as control). At 48 h post transfection, the dual-luciferase reporter assays were performed to calculate the relative ratios of firefly and renilla luciferase activities using the Dual-Glo^®^ Luciferase Assay System kit (Promega, USA) according to the manufacturer’s instructions. All experiments were repeated for six times.

### Confocal laser scanning microscopy

At 12 hours before the transfection, Drosophila S2 cells were seeded onto cover slips in a 24-well plate (TPP, Switzerland) with approximate 40% confluent. S2 cells of each well were transfected with 0.5 μg pAc5.1-LvFLp53-GFP or pAc5.1-LvΔNp53-GFP plasmid using the FuGENE HD Transfection Reagent (Promega, USA). At 36 h post transfection, subcellular localizations analysis were conducted using the Hochest 33258 Solution (Beyotime, China) and visualized with confocal laser scanning microscope (Leica TCS-SP5, Germany).

### DsRNA production, RNAi performance and AMPs detection with *V. parahaemolyticus* challenge

According to the sequence differences, we designed two pairs of primers (Table 1) to produce two dsRNAs, which can specially target to LvFLp53 and LvΔNp53, but not interfere with each other. The synthesis of dsRNAs including dsRNA-LvFLp53, dsRNA-LvΔNp53 and dsRNA-GFP (as a control) were performed with T7 RiboMAX™ Express RNAi System kit (Promega, USA) with the user’s manual. The length of dsRNA-LvFLp53, dsRNA-LvΔNp53 and dsRNA-GFP are 466 bp, 168** **bp and 554 bp, respectively. The experimental group was treated with the injections of dsRNA-LvFLp53 and dsRNA-LvΔNp53 (10 μg dsRNA each shrimp in 50 μl PBS), while the control group was injected with equivalent dsRNA-GFP (positive control) and PBS (negative control), respectively. Forty-eight hours later, shrimps were injected again with 1 × 10^5^ CFU *V. parahaemolyticus*, and mock-challenged with PBS as a control. The shrimps cultured in tanks with air-pumped circulating seawater were fed with artificial diet three times a day at 5% of body weight for about 2 days following infection. The hemocytes from each group (9 shrimps) were sampled for real-time RT-PCR to detect the knockdown efficiency of LvFLp53 or LvΔNp53 and expression levels of AMP genes. Primer sequences were listed in Table 1 and Supplementary Fig. 4.

### WSSV challenge experiments in LvFLp53 and LvΔNp53-knockdown shrimps

The synthesis of dsRNAs was the same as described above. Healthy *L. vannamei* (average 4~6 g, *n* = 40) received an intramuscular injection of 50 μl LvFLp53 dsRNA, LvΔNp53 dsRNA or GFP dsRNA (10 μg dsRNA per shrimp) or PBS only. Forty-eight hours later, shrimps were injected again with 1 × 10^5^ copies of WSSV particles and mock-challenged with PBS as a control. Shrimps mortality rates were scored on an every 4 h basis over 6 days. Differences in the cumulative mortality between treatments were tested for statistical significance using the Kaplan-Meier plot (log-rank χ^2^ test) using the GraphPad Prism software.

A parallel experiment was also performed to monitor the WSSV replication in LvFLp53 and LvΔNp53-knockdown shrimps (*n* = 150 each group). Briefly, three samples of muscle and hemocytes (each sample pooled from 3 shrimps) were collected from each group at 24, 48, 72 and 120 h post infection. Muscle DNA was extracted with TIANGEN Marine Animals DNA Kit (TIANGEN, China) according to the user’s introduction. The viral loads were measured by utilizing absolute real-time quantitative PCR with primers WSSV32678-F/WSSV32753-R and a TaqMan fluorogenic probe (Table 1) as reported in previous study^49^. The WSSV genome copy numbers in 0.1 μg of shrimp muscle DNA were then calculated. The cDNAs from the hemocytes were generated with the same method above. The WSSV gene VP28 expression was detected by using the real-time RT-PCR with the primer VP28-F/VP28-R (Table 1).

An additional experiment was also performed to investigate the effects of knockdown of the two p53 isoforms by RNAi *in vivo* on the expression of LvDorsal. In brief, at 48 hours post injection of LvFLp53 or LvΔNp53 dsRNAs, the gills and hemocytes of shrimps were harvested. The nuclear and cytoplasmic fractions of hemocytes were collected according to the protocol of NE-PER Nuclear and Cytoplasmic Extraction Reagents (Thermo, USA), and then identified by immunoblotting with Histone H3 (D1H2) XP Rabbit mAb antibody (Cell Signaling Technology, USA) and HSP90 Rabbit polyclonal Antibody (Proteintech, USA), which used as the internal references of nuclear and cytoplasmic extracts, respectively. The detection of LvDorsal was performed by Western blotting using prepared rabbit anti-LvDorsal antibody from our previous study^50^. Besides, the total RNAs from gills and hemocytes were isolated and real-time PCR was performed to detect the expression of LvDorsal mRNA (Table 1).

### Co-immunoprecipitation and western blot

To explore the potential interaction between LvFLp53, LvΔNp53 and LvDorsal, pAc5.1-LvFLp53-FLAG or pAc5.1-LvΔNp53-FLAG was transfected with pAc5.1-LvDorsal-GFP or pAc5.1-GFP (as a control) into S2 cells. After 48 h, cells were harvested and lysed in Pierce IP lysis buffer (Thermo, USA) with proteinase inhibitor cocktail (Sigma, USA). The co-immunoprecipitations were performed using anti-FLAG tag agarose conjugated gel (Abmart, China) and anti-GFP tag agarose affinity gel (MBL International Corporation, Japan), respectively. Western blotting was performed with rabbit anti-GFP antibody (Sigma, USA) and rabbit anti-FLAG antibody (Sigma, USA), and alkaline phosphatase-conjugated goat anti-rabbit secondary antibodies (Sigma, USA). A standardized aliquot (5%) of each total input cell lysates was also examined as control.

## Additional Information

**How to cite this article:** Li, H. *et al*. Identification of two p53 isoforms from *Litopenaeus vannamei* and their interaction with NF-κB to induce distinct immune response. *Sci. Rep.* **7**, 45821; doi: 10.1038/srep45821 (2017).

**Publisher's note:** Springer Nature remains neutral with regard to jurisdictional claims in published maps and institutional affiliations.

## Supplementary Material

Supplementary Information

## Figures and Tables

**Table 1:** Summary of primers and atificial sequences in this study.

Name	Sequence (5′–3′)
RACE	
Lvp53-3RACE1	AATAGAGTATGAAGCTGAGTGAAG
Lvp53-3RACE2	TGACGCGCTCATGTATCCTGAAGAG
Lvp53-5RACE1	TAGATCCTGACTGTAGCACCACTTG
Lvp53-5RACE2	CAGAGTAGAGTCAGCCAGGCAGGAA
Genomic DNA amplification	
GLvp53-F1	GCGGGATGCAGCGGTCGGACTCC
GLvp53-R1	GATCATCTTGGCTTCGGTGGCGACGA
GLvp53-F2	AGTCGTGTTTTTTAGTCTTAAGTC
GLvp53-R2	CAGGAAGTGGTAGAGCCTTCAATCA
GLvp53-F3	AAGCAATGATCATCGTCGATAGGTT
GLvp53-R3	CATGGTGATGTTAGCATTCACCCAG
GLvp53-F4	GCAAACTCTACCTCTGCCCAAATG
GLvp53-R4	CAACGCATGAAGTCAGACACATAAT
GLvp53-F5	GCATTTAGTGCAGGTCGAGGGTG
GLvp53-R5	GATACATGAGCGCGTCAGGCTGCCA
GLvp53-F6	GTCCCAATAGAGTATGAAGCTGAGG
GLvp53-R6	ATTGTCAACCTTTATTCAAAATAATAT
Real-time RT-PCR	
LvEF-1α-F	TATGCTCCTTTTGGACGTTTTGC
LvEF-1α-R	CCTTTTCTGCGGCCTTGGTAG
LvFLp53-F	ACTCTGCCAAGAAACGCCCTC
LvFLp53-R	ATCCCTGAGCAGGTGATACTCG
LvΔNp53-F	AGAAGGGCAACTCCGTCGTG
LvΔNp53-R	TATCGACGATGATCATCTTGGC
VP28-F	AACACCTCCTCCTTCACCC
VP28-R	GGTCTCAGTGCCAGAGTAGGT
LvDorsal-F	TGGGGAAGGAAGGATGC
LvDorsal-R	CGTAACTTGAGGGCATCTTC
WSSV32678-F	TGTTTTCTGTATGTAATGCGTGTAGGT
WSSV32753-R	CCCACTCCATGGCCTTCA
TaqMan probe-WSSV32706	CAAGTACCCAGGCCCAGTGTCATACGTT
Protein expression	
FLAG coding sequence	TTCGAAATGGAAGACTACAAGGACCACGACGGCGACTACAAGGACCACGACATCGACTACAAGGACGACGACGACAAGTAAGTTTAAAC
LvFLp53-F	GGGGTACCATCAAAATGCAGCGGTCGGACTCCGAG
LvΔNp53-F	GGGGTACCATCAAAATGATCATCGTCGATAGGTTGC
Lvp53-R	TTGGGCCCGTTACTCTCCTCTTCAGGATACATGAG
LvDorsal-F	GGGGTACC ATGGCTGACCCAATGTTTGTT
LvDorsal-R	ATAGTTTAGCGGCCGCCACATATCAGAAAATATCCAAAACTTACC
dsRNA templates amplification	
dsRNA-LvΔNp53-T7-F	GGATCCTAATACGACTCACTATAGGAGAAGGGCAACTCCGTCGTG
dsRNA-LvΔN p53-R	CTATCGACGATGATCATCTTGGC
dsRNA-LvΔN p53-F	AGAAGGGCAACTCCGTCGTG
dsRNA-LvΔN p53-T7-R	GGATCCTAATACGACTCACTATAGGCTATCGACGATGATCATCTTGGC
dsRNA-LvFLp53-T7-F	GGATCCTAATACGACTCACTATAGGATGTTGGTGGAAGTGTTGGATTTG
dsRNA-LvFLp53-R	CAGGTGCTGTTGCTGTGAAGTGT
dsRNA-LvFLp53-F	ATGTTGGTGGAAGTGTTGGATTTG
dsRNA-LvFLp53-T7-R	GGATCCTAATACGACTCACTATAGGCAGGTGCTGTTGCTGTGAAGTGT
dsRNA-GFP-T7-F	GGATCCTAATACGACTCACTATAGGCGACGTAAACGGCCACAAGTT
dsRNA-GFP-R	ATGGGGGTGTTCTGCTGGTAG
dsRNA-GFP-F	CGACGTAAACGGCCACAAGTT
dsRNA-GFP-T7-R	GGATCCTAATACGACTCACTATAGGATGGGGGTGTTCTGCTGGTAG
