# Supplementary material for: Identification of two p53 isoforms from Litopenaeus vannamei and their interaction with NF-κB to induce distinct immune response
Source: Sci Rep. 2017 Mar 31;7:45821. doi: 10.1038/srep45821 (PMC5374463; doi:10.1038/srep45821)
Supplement: Supplementary Information [file srep45821-s1.pdf]

Identification of two p53 isoforms from *Litopenaeus vannamei* and their interaction with NF-κB to induce distinct immune response

Haoyang Li [1, 2, 3], Sheng Wang [1, 2, 3], Yonggui Chen [1, 2, 3, 4, 5], Kai Lǚ [1, 2, 3], Bin Yin [1, 2, 3], Sedong Li [6], Jianguo He [1, 2, 3, 4, 5, *], Chaozheng Li [1, 2, 3, 4, 5, *]

1. MOE Key Laboratory of Aquatic Product Safety / State Key Laboratory for Biocontrol, School of Life Sciences, Sun Yat-sen University, Guangzhou, P. R. China
2. Institute of Aquatic Economic Animals and Guangdong Province Key Laboratory for Aquatic Economic Animals, Sun Yat-sen University
3. Guangdong Provincial Key Laboratory of Marine Resources and Coastal Engineering
4. School of Marine Sciences, Sun Yat-sen University, Guangzhou, P. R. China
5. South China Sea Resource Exploitation and Protection Collaborative Innovation Center (SCS-REPIC)
6. Fisheries Research Institute of Zhanjiang, Zhanjiang, P. R. China

* Corresponding Author:
Chaozheng Li, Ph.D.
School of Life Sciences, School of Marine Sciences
Sun Yat-sen University
Guangzhou, 510275
P. R. China
Tel: +86 20 39332850;
Fax: +86 20 84113229;
Email: lichaozh@mail2.sysu.edu.cn

* Corresponding Author:
Jianguo He, Ph.D.
School of Life Sciences, School of Marine Sciences
Sun Yat-sen University
Guangzhou, 510275
P. R. China
Tel: +86 20 39332988;
Fax: +86 20 84113229;
Email: lsshjg@mail.sysu.edu.cn

Supplementary Fig. 1. Comparison between the cDNA of LvFLp53 and LvΔNp53. The full-length cDNA sequences and deduced amino acid sequences of LvFLp53 and LvΔNp53 were shown in Supplementary Fig. 1A and Supplementary Fig. 1B, respectively. Nucleotides and amino acids were numbered on the left of the sequences. The ORF of the nucleotide sequence was shown in uppercase letters, while the 5' and 3'-UTR sequences were shown in lowercase. Amino acid sequence was represented with one-letter codes above the nucleotide sequence. The differences between the N terminal sequence of LvFLp53 and that of LvΔNp53 were underlined and the potential poly A signal (aataaa) was boxed with black line.

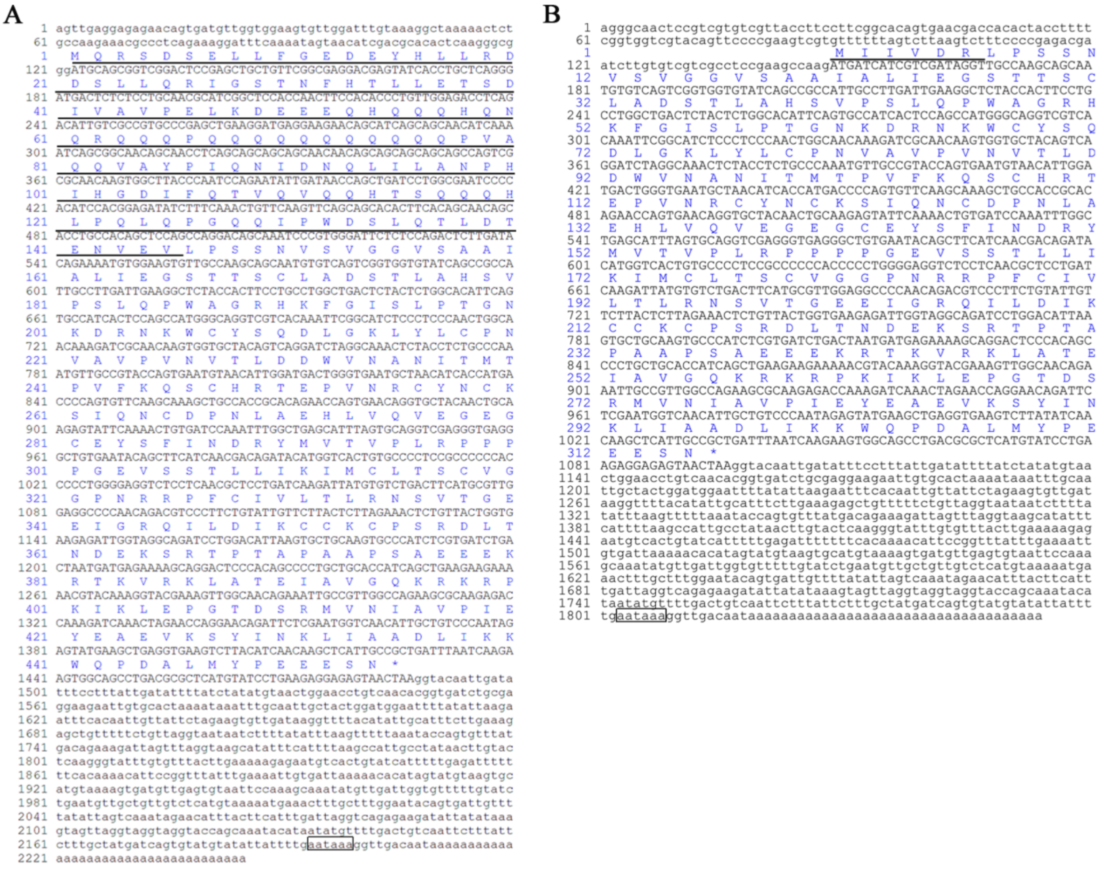

Supplementary Fig. 2. The mRNA levels and the protein levels of LvDorsal responsed to the konckdown of the two p53 isoforms. (A) The mRNA levels of LvDorsal in gills (A1) and hemocytes (A2) of the LvFLp53 or LvΔNp53 silenced shrimps. (B) Subcellular distribution of the LvDorsal protein in hemocytes with the treatment of LvFLp53 or LvΔNp53 dsRNAs.

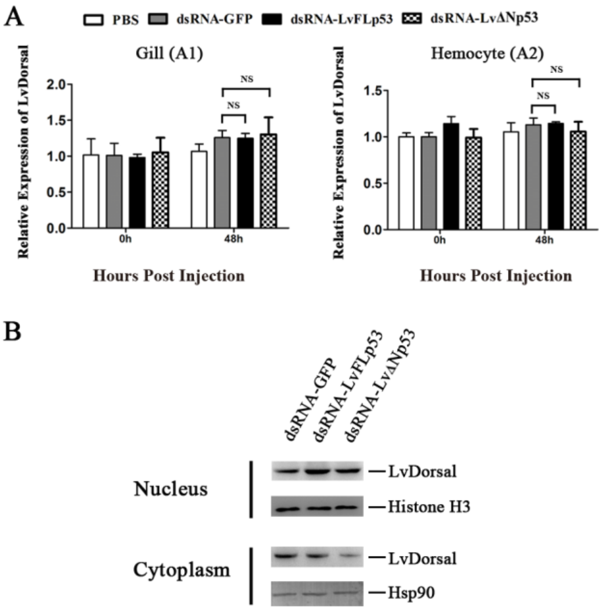

Supplementary Fig. 3

5' flanking regions of reporter gene plasmids including *Drosophila melanogaster*, *Litopenaeus vannamei* and *Penaeus monodon* antimicrobial peptides. GenBank accession numbers are showed and the location of primers are underlined.

>*Litopenaeus vannamei* PEN2 (LvPEN2) DQ206401

AAAAAACTAGTTCCTTATTTTTATTTTATCGATAATAGCAAAATATTGTTAATGTTA
ACTGCTGTAACTTTGTTTTAGTATAAATACACCCTATAAATTTGTTGAGGTAGTTGT
ATTTGGGCTCAAATAAGCGACTACTGGAAATGTTACGGTCCTGGCCCCGGGGTCGTT
GCCTGTCGGCGGTCCTGCATATACATATACATACATACGACGCCTGAAGGTGCTTTC
ACAACCGCGTGGCTTCTCCATAAAAGGCATGGCACCACGGCCTCCGGTGCCACTCGG
CGCTTGGCTCTCCCTCGAGCCTCACCTGCAGAGACCGACGCTCCGAGCCCGGGTTCC
CTCCTGCG

LvPEN2-F: GGGGTACCACTAGTTCCTTATTTTTATTTTATCGAT
LvPEN2-R: GGAGATCTCGCAGGAGGGAACCCGG

>*Litopenaeus vannamei* PEN3 (LvPEN3) DQ206403

GACTACTGGAAATGTTTACGGTCCTGGCCCCGGGGTCGTTGCCTGTCGGCGGTCCTG
CATATACATATACATATATACGACGCCTGAAGGTGCTTTCACAACCGCGTGGCTTCA
CCATAAAAGGCATGGCACCACGGCCTCCGGTGCCACTCGGCGCTTGGCTCTCCCTCG
AGCCTCACCTGCAGAGACCGACGCTCCGAGCCCGGGTTCCCTCCTGCGTCCGCC

LvPEN3-F: GGGGTACCGACTACTGGAAATGTTTACGGTCCT
LvPEN3-R: GGAGATCTGGCGGACGCAGGAGGG

>*Litopenaeus vannamei* PEN4 (LvPEN4) DQ206402

ACATGCAGATACAGATACATATATTCATATTTATATAATAAGTATGTATTTATCTAC
CCATGCCTTTATATTTACAATAATAGATGCCTATATGTATGCGAGTCAGAATAGAGG
GCAACCATAACGGAAACACACAACGCTCATCGGGTCATTGCAATCTGTTTGAAAACT
TCCCTAAAGACGATCGCTAACAGTGTGACTATTGTGTAGTTTTAGGTGATCTTCATT
ACGAGTGTTATTTATGTTTAGAAAATAAGGGGTTTTAGATATCAAAATTGATGATTA
ACAAGGATTCACTCGAAAAAATTATTTCATTTCTACCCTCTGTTCATTTGTTTATTT
GTCTCATAGTCTGTTTAGCTATTTATTTCTCAGCCTGTCGTTCTGCTTAGCTACGTC
ACTTTATCTATCTGTCTCTCTTATCTCTCTTATCCCCCTTCTCTTTCTTTCTCCTAC
CCAGTTTCCCGTTTTCCCTTCCCTCCTATGCATCCTTTTTTCTCTCTCCCTTATGCC
CTTTCGCTCTCTATATCTCTTCCATCCTGTCCTTTCTCTCTCCCAAGTTGCTTGCAC
AACCGCGCGGCGTCTCCATAAAAGGCATGGCACCGAAGCGTCCGGTGCCACTCGGCG
CTTGGCTCTCCCTCGAGCCTCACCTGCAGAGACCGACGCTCCGAGCCCGGGTTGCCT
CCTGCGTCCGCC

LvPEN4-F: GGGGTACCACATGCAGATACAGATACATATATTCATATT
LvPEN4-R: GGAAGATCTGCGGACGCAGGAGGCAAC

>*Penaeus monodon* Type411 (PmPEN411) FJ418752.1

CATTTACATGAAATTGAAAAGAACTGGACATCGTTTGAAAACCCACTAGATTCTCCC
TCTTTTCTCGTTCTCTCTGTCTGCCTTCCTGTTTGTCTGTCTGTCTGTCTGTCTCTC
TCTCTATATATGCTATATATGTATACATATGTATACTTCAAAGACCTTACACATCTC
AATATATATGCATATATATGTGTGTGTGTGTGTGTGTGTGTGTGTGTGTGTGTGTGT
GTGTGCATGTGTGTATAGATGTATGTAAAGCATGGAAAACGACTCAAGGTGCTTGCA
CAACATCGTGGCGTCTCTATATAAGCCAGCCACCTCAGCTTCCAGTACCAGTCGGTG
CTTGGCTCTCACCTGACCCCCACCTGTAGAGGCCGAGACTCCTTGCCCGGGTTCCTT
CCTGTGTCCGCCATGCGTCTCGTGGTCTGCCTGGTCTTCCTGGCTTCCTTCGCCCTG
GTCTGCCAAGCCCAAGGGTACCAGGGTGGTTACACACGCCCGTTCCCCAGACCACCC
TATGGGGGAGGATATCATCCAGTTCCTGGTAAAGTTTCTAAAGGTTATTTGTTGGTC
TATATGGTAATCGTATTATTGTCACCAATCACATCATATTGTTATTAGTAATCGCTA
ACATTATTTACATTCCATATTTATTACTATTACTGAATATCTCTATTCCTTCAATAC
AACTATTCATTCTGTGTTAAAAAATGTATATAGTTTATTGTTAGTATTGACAACATT
TAAAAGAGATGCAGATGATACTCGTAAAAGAGAAAGAGAGAAAAAAACATAATGATG
ATTAAATAATGATAGCTTTAATGATAACCATGAGTCTACTGTCTTACCAA
PmPEN411-F: GGGGTACCCATTTACATGAAATTGAAAAGAACT
PmPEN411-R: GGAAGATCTTTGGTAAGACAGTAGACTCATGGTT

>*Penaeus monodon* Type536 (PmPEN536) FJ418753.1
ACCCCCTTTGAGAACTCTCCTGACAACGCTTACTAACAGCTTGCCTGTTGTATAGTG
TCAGATGGTCTCCATTACGTGTGGTATATGTTTAAAAAAAAAGGGAGGTTTAAAAGT
TAAAATTGATGATGATGATGGTGAATAATGATGAAACTGAAAAATCTTATATTTTTT
CCATGTTTTTTATCTGTCTGTCTGTTAATCTAATAGTTAGCTATCTATCTATTTACC
ATTCTGTTTAGTTTTGAGTCTCTTTTTCTATTTGTCTCTATGCCTATTTATATTTCT
TTCTTTCCCTCTTACCCCCCTTCCCTCTCTCTCTCTCTCTCTCTCTCTCTCTCTCTC
TCTCTCCTTTCTCTATCTATCTCTACCTTCCTGTCTTTCTCTCCCCCCGTCTCTCTC
TTTAAAACTGCTTGCACAACCGCGTGGCGTCTCTATAAAAGCACCACAGCCCCCGGT
GCCAGTCGGTGCTTGGCTCTCACCTGACCCCCACCTGTAGAGGCCGAGACTCCTTGC
CCGGGTTCCTTCCTGTGTCCGCCATGC
PmPEN536-F: GGGGTACCACCCCCTTTGAGAACTCTCCTGACA
PmPEN536-R: GGAAGATCTGGCGGACACAGGAAGGAACC

>*Drosophila melanogaster* Metchnikowin (Mtk) AAF58139.1
ATTTAAAGGGTAGCGCCACGTTCAACCTCTTTTGCAGCCCCATTCTGCTGCGAGAAA
ACTAACAAAGTGCTCTAATCGAGCCAAGGGGCAATTTCTTGTGTTGCTGCAGCTGCA
CTTTGCACCTCCGCATCCGTGCACCCAAAAACCCGCTTTCTAGATGTTCTCATCATG
CACTGAAAAAGAATCCAAATTTTTACAAGAAATAGTTTAAAATTAGGTAATGTGAAA
GATATCGGCACACGGACAGGCCGAATTATCTGTTGTAAAACTAGCTGCTCAGTTATT
AAAAACATTTGTAGTTGCTGACGTTTCCATACAGAGACTAATTTTATTTTCACGGAC
AGGGGTTTTCCGCTTTAATTGCTTCATTTTTGTTGCTTTATTGCGTGTATATTGCCC
CACAAAACAGATATAAATCATTCGCGCATATCGTAAATGTTGGTAGAAAATGACAAA
CAAGAGAAAAAAGATGATTAAAAGCTTCAAGACAATCCTCTATAGGATCTGATTAAA
TATGAATATTTTATTTATTAGTTTTCTTTCTGTGTACGGCTTAGAAGGCAGAAGCTG
CGAGGGGCGTAGGGCAGTGGGCGTGGCTCCGTGTTGACGCATGTTGACTATGCCTTT

GAATGGCTGCCGTGGTTGTCGGTGGGTAATTTGCAATGCAGAAAAACCAACAGGGCG
CTAAAAAGGAGAGTGTTTTCGTGGGAGGTGGAGATGGTCACTGGGGGCAACATAAAT
ATTCAGCGAGAAACGTCATATTTACATTTAGTCTAGGCTGATAATCCGGGACCGTGG
GAAGTCCCCTTTGGGTGGTGCTGGCTGGGTTCCCCTGGCCACAATCGGTTATCTGCC
CCCGGCTGACACTTGCCCGTCATTCATTCGGCTGCTTATCGCAGAAGCTCAAATAAA
AAGTCCCCAATCTGCGACTCGTTTGTCTGGGACTGAGCTATAAAAGCCTCACCATCT
CAACGCTCAAAGCATCAATCAATTCCCGCCACCGAGCTAAG

DmMtk-F: GGGGTACCATTTAAAGGGTAGCGCCACGTTC

DmMtk-R: GGAAGATCTCTTAGCTCGGTGGCGGGAATTGATT

>*Drosophila melanogaster* Cecropins A (CecA) AAF57025.1

GAAAAACAACTAAGTTACTAACGCAAGACTTTTAGTTAAGTTAGTTAATATAGACCG
AGATGTATGTACATACATACCGCTTTCGCTTACAATAAAATGTTAAATAAGTTTTCA
GATTCGTACGTGCTCAGTAAACAATTATTTTTTATTGTCATTTAATGCCTATTGAAT
TTTTCAAACTTAATTTAGTGCCTTTAGTAAAATATTGTAGTGATTCCCCTCGAAAAA
TACCACAAATTGGATGCGTTTATGTAAATAAATTGCCCTTGAGTGATAGAGTAAATT
TGAATTTGACTGTCTTAGAAAGATAGAAAGAGATCAATTCAAAATGCCAAAAGGATA
GAGTTATTAAAGCTCTAATTCAAATTGGCCCAGAACCGTTTAAAGGATATTACAATT
TGTAATTTACATATTTGGATTATAGCATTGAAATCCCCGATTGTTCCCTAGATGTGC
AGATGTGTGCTTGGAATCAGATCGGTTACCTTCAGTGTACTTTTCTCTGCAAAAATC
CCCGTGCATGCCTTATCTGTCATTTTGTTTTTCAAGCTGGCTGTTCGCCTATAAAAG
CTCTCGCCTTTTGTATCGCAGTCATCAGTCGCTCAGACCTCACTGCAATATCAATAT
CTTTAGCTTCTCCTAAGAAAAAATCAAGAAAATATCACC

DmCecA-F: GGGGTACCGAAAAACAACTAAGTTACTAACGCA

DmCecA-R: GGAAGATCTGGTGATATTTTCTTGATTTTTTCTT

>*Drosophila melanogaster* Drosomycin (Drs) AJ885064.1

CAATGAAAGTGATAATACGAATTGACCATGTAGCAATTTGTTTTGTGTCTATAGACT
GAATTTTTTCCCCTACATTAATCAAAATTATATTTTTATATTTTATTTTGAGTTTAC
TTGGGTTTTTCATGAAATTAAAGATTAACCTGGGGTTTTTACAATCCATTACGATAG
GCTTTCTGTTCATTGCATCTATAGCCTCTGTACTTTTCCGTGCATTCTTAAAGCAAA
AGCATACATGTATATCTTCAATTCAAGTATATCTGCAATTTAGTTTGCTTATCTGGA
GTCGCTGTATCCCGACCTATCCAAACTCGCGTCCCAGTCAAAGGTAAACCATTTTTA
TTTAGTTCCCAGCCTCTGGTATTTGTTGTTATTTATCGGTGACTTTTTGAAATTTAT
TCAATTAATTTCAGTCTTTGTGCTCTTGATAACACGATTTCCTCGTTATTCATTTAG
TTTGGGTTTAACCAAAACCCTTTAGCGAATCATTTTTGCTGGACAGTCCAGTTGAAT
TCGGTATTCTACACACAAAGCTCATCTTACAGTGAAAAGTGTTACTTTATGAAATAC
AAATGAGGCTCTAAGCAATGCTTTTCGCTTACGCTTTTCGATAAGCGTACAAGTAGT
TCCCCTACCGAAGGCCTATAAATGTGACTGCACATGTATCATCATAATTTGTTGATA
TACTTCGTTTATACCCGACTACGCATCGGCTAAAGCTGAGGGATCGGTGCACTATAT
AAGCTTCTCCTCGAAGTTCCCAAGCCACAAGTCGCTGATAATTCAAACAGAAATCAT
TTACCAAGCTCCGTGAGAACCTTTTCCAAT

DmDrs-F: GGGGTACCCAATGAAAGTGATAATACGAATTGACC

DmDrs-R: GGAAGATCTATTGGAAAAGGTTCTCACGGAG

>*Drosophila melanogaster* Attacin A (AttA) AY056895.1

GCCATCAGGCCACCACCCATTCTGCCCGCCTAAAGATGTGTGCATACCGCGGAGAAG
TCATCCGATCAAATTTGTTTTGAAAAATCTTTATAAAAATTGTGAATTTTTTACTTT
CTGCAAACAGTAAGCAATAAACACACGAAAGACAGCAATTAATAATCTTCAATCAAT
TGTGACACAATGAGGGGTTCCCATCGCTTATCAGCGGTTTTTGTACCGAATCTGCTG
AGCTCTAGAGCTGATAAGAAATATACTTGCTCAAAACAAAACCACAAAAGTCACGTT
TGAGAGAAAAAAAGCCTAAACGAATTTAATTCGCGACTCATATGAATCACAAACCTG
TTCTATAGCACGTTCTTCTTAAATTCTAGCGAAATAACCAGATGGCTGCAAATCATA
ATGAATGGGTTTGTCCCCTAAAAAAAACGAACTGACAAGCCCCCTTATAAAACTTAT
TTATTAATAGATTAGTTCGTATATAATTGCATATGTAAATACTTTAAATAGAACAAA
TTATTACGACATTTAAAAATATATATCCTGGTTTTTAAAAACAGGGTTTGAAAAAAA
TGTTTATAAGCTAATTACCTGGTAGATTATATTTTTCAGTGCAAACTTTTCGTTGAC
ACTGCGGGTTAAAGTTTCCGCTCTCCTCTCTTTCGGCTCGCATCCTTTTTGCCCGCT
CCTGCGCAGAAAATTCAATTAGGATTCGGCTGAAACTTCACTCAAATCCTGCCGCTC
TCACCGTCGCTCTTTATTTCGCTCGCCTTCCCTTTCCGGCTCCCCCAGAATAATCCC
TCCACGCAAAATAACGTATTGATAAAGCCTGACATCAAGTGAGAAATACGATAGAGA
ATCCCTTATAACTGTCAAATGCCGACCTGCGCAAGATGAGGATGCACTCCTCCATCA
AGACACAAAAGAAACCACTTGGAGACGCTGACAGAGGTTCTGCGGCGAGGGTGAAAC
TGGACAATGCAGCAACAAGTGGCGTCAATGGGTCGCAAAAAAGGGGAGGTGATGAGG
TCAAGTCGCACAGCCACACAAGCAAACAGCAGAAGTAAACCACCATCACATCTGAGC
GGGGAATTTCGCTTTGATAAGGCATCCAGGCCGAGATCGGCAATCAGATGAATCATG
TCAATCATCAGAAAAGTTCTTCCCCGCATCTTGAGGTATAAAACCGATGCATTGGAC
ACCTTGAAACATCAGTCAGCTCCAGCAATCCAGTTCAGCAAC

DmAttA-F: GGGGTACCGCCATCAGGCCACCACCCATTCTG

DmAttA-R: GGAAGATCTGTTGCTGAACTGGATTGCTGGAGCTGAC

>*Drosophila melanogaster* Defensin (Def) AAF58855.1

TGACGCCAAAATGCAAGACAAGACAACCTGGTCGATAACAAAGGTAAACAGGCAACG
GCCAGCCAAGGAGCAGGGCAACTGAAAAAGCCTCGGTCGTGGGATTCGATCGGGATT
GTCGGCTCAGCGCGACTGGGCTGAGAACCAGATGCAGATGCCGATACAGATATAGAT
ACACGTACGCGCAGATACGGATTCAGATACAAGTACACCCGCCCCTGCCGCTGTATG
CCCAACTAATCATTGTGTGATTCTTGTTTGTTTATTTGCCCGGCATTATGAAGAGAC
TTTTCGGTAGAAATTATTTATTGTCGCATGTGTTTATGTATCCGTAACCGAGTATCT
CAGTTGCTTGAGCCAACTGTGTAGCTGTGTAGCTGTGAGTATAGCCCTTAAAGTGGC
ACCCAATCGGTCAGTTAGCTAGAAATTCAGATGATTAAATATGGATTCCCCTACATC
AGCTAATTTCAACAGTTTGGGAGTAATAAAATCGAAATTGGATGCTACTAAAGGGCA
CATATTTACTTAGGCTTTTATCAACGTTGCATATATACAAATATCCTGCATATTTCG
CAAACCAAAGATTCTTTCTCAAGTAAGGCCTAAACAATTTGAAATGGTTAATTTCGT
AGATGTTGCTTTTTACAATTAACTTGTCATGTGGAATATACTTTACTGCCTAAAATT
TAAGGCAGTTAAAATCCCTAGAAATGCAAATAACTTATTGCAGAAACGGGCTCTGTC
GGCTGTATTTTGCTCTTATCTATGAAATATTGTCAATATTTTCCAGGCAAAGCACAT
GAAATAATGATCTAGACAACGGTTTCTCCCATTTGCAGTGAACTTAAAAATTAAAAA
CCCCCGAGACGTGTCTTCCTGCACAGAAAAAGAGACAATGGGAAGGTAAGTCACCGG

GTGGGAGTCCCTGGGCCGAATCGATCAGCCCGTCGCATTGCTATATAAGCTCGGCGA
AACCACAATCTGCAACAAC<u>AGTATCTCTCCAGTTGTATTCCAAG</u>

DmDef-F: GGGGTACCTGACGCCAAAATGCAAGACAAG

DmDef-R: GGAAGATCTCTTGGAATACAACTGGAGAGATACT

Artificial promoters from NF-κB activating element luciferase reporter plasmid

AGGAATTTCCAGGAATTTCCA

Supplementary Fig. 4

cDNA sequences and real-time RT-PCR primers of *Litopenaeus vannamei* AMPs including LvALF1, LvCrustin1, LvLYZ1 and LvPEN2. The GenBank accession numbers were showed and the location of primers were underlined.

>LvALF1 EW713395

AGTCGGCGACGGACAGGCTTCCGAGCAACACCGCTTCCGCATTCGGCCTTGACTTCG
GGGGGAAAAGACGACGATGCGGGTGCTGGTCAGCTCTGTAGTGGCACTCGCCCTGAT
TGCTCTTGTGCCACGGAGCCAGGGTCAGGGAGTGCAGGACCTCATCCCTTCGCTAGT
CCAGAGGATAGTCGGGTTGTGGCACTCGGATGAGGTGGAGTTCATGGGTCACAGCTG
CAGGTACAGTCAGCGGCCCTCCTTCTATAGGTGGGAGCTTTACTTCAATGGCAGGAT
GTGGTGTCCTGGATGGGCTCCCTTCACTGGCAGATCTCGCACCCGCAGCCCTTCCGG
CGCCGTCGAGCACGCGACGAGGGACTTCGTGCAGAAGGCGCTGCAGAGTAATCTCAT
CACGGAGGACGACGCTAGAATTTGGCTCGAGCACTAAGGCCTTTGTCTCAAGGTCAT
TCCCATTTCCTTTTGACGCGATGAAGGTCGAAGCGATATTTGTAAATCGTGAATAAG
AAGAATGATGTCAGC

LvALF1-F: TTACTTCAATGGCAGGATGTGG
LvALF1-R: GTCCTCCGTGATGAGATTACTCTG

>LvCRU1 AF430071.1

ACCACTAGCTTGTACTGGAGGCAACCATGAAGGGCATCAAGGCGGTGATTCTGTGCG
GCCTCTTTACGGCGGTTTTGGCTGGCAAGTTTCGCGGCTTCGGACAGCCATTTGGAG
GTCTGGGTGGTCCAGGAGGCGGTGTAGGTGTTGGTGGTGGTTTCCCCGGAGGCGGTT
TAGGTGTAGGTGGCGGTCTTGGTGTAGGTGGCGGTCTTGGTGTGGGTGGCGGTCTTG
GTGTAGGTGGCGGTCTTGGAACTGGCACAAGCGACTGCAGGTATTGGTGCAAGACTC
CGGAGGGTCAAGCCTACTGCTGCGAGTCGGCCCACGAACCAGAGACACCTGTTGGCA
CCAAGCCACTCGACTGCCCACAAGTCCGTCCCACATGCCCACGTTTCCATGGGCCCC
CTACAACCTGTTCCAACGACTACAAGTGTGCTGGCCTCGATAAGTGTTGCTTCGACA
GGTGTTTGGGAGAACACGTGTGCAAGCCTCCCTCATTCTTCGGATCGCAGGTTTTCG
GATGAAGGATAAGCACGAAAGAATTTGAAAGGATGAAGAGAAAGAAGAAAAGACCAT
CTGAAGAACGACCGATGTTTTGGAATTTGACTGAAAAAAGAAAGAAAAACAGGGAAT
TCTTTCTTTCTGTAGGATTTATCTGATTACCATGATTTTTTTTATTTGTGAATTAGA
CTATTCTTCTGTCAAAAGAAACTTATAGGCC

LvCRU1-F: GTAGGTGTTGGTGGTGGTTTC
LvCRU1-R: CTCGCAGCAGTAGGCTTGAC

>LvPEN2 DQ206401.1

AAAAAACTAGTTCCTTATTTTTATTTTATCGATAATAGCAAAATATTGTTAATGTTA
ACTGCTGTAACTTTGTTTTAGTATAAATACACCCTATAAATTTGTTGAGGTAGTTGT
ATTTGGGCTCAAATAAGCGACTACTGGAAATGTTACGGTCCTGGCCCCGGGGTCGTT
GCCTGTCGGCGGTCCTGCATATACATATACATACATACGACGCCTGAAGGTGCTTTC
ACAACCGCGTGGCTTCTCCATAAAAGGCATGGCACCACGGCCTCCGGTGCCACTCGG

CGCTTGGCTCTCCCTCGAGCCTCACCTGCAGAGACCGACGCTCCGAGCCCGGGTTCC
CTCCTGCGTCCGCCATGCGCCTCGTGGTCTGCCTGGTCTTCTTGGCCTCCTTCGCCC
TGGTCTGCCAAGGCGAAGCGTACAGGGGCGGTTACACAGGCCCGATACCCAGGCCAC
CACCCATTGGAAGACCACCGTTCAGACCTGTTTGCAATGCATGCTACAGACTTTCCG
TCTCAGATGCTCGCAATTGCTGCATCAAGTTCGGAAGCTGTTGTCACTTAGTAAAAG
GATAAAGAAATTGACGGAGAAGACAATGGAAACCTGGCTTGACAACTTGTTAATTAA
TACTCATATGTGAAGAGATTGCAACCCTGATTTTGAGCTGTATTTTCTCGTTCAATT
TTGTTTACTTTTGCTTGTGGAAAGGATGTGGGTATTTCGTCTATCCATCGCTAAAGA
TTCTTCCATGAATGTATGATGAAGGAAAGTGCATGTGTGTAAGTATGTATGTATGTG
CTTACAGGTATTTGTTGCATTAAGTGTCCGTGTATTTAGGATCTGCAACACACGAGG
AAGAGAATATTTGCCA

LvPEN2-F: GACGGAGAAGACAATGGAAACC

LvPEN2-R: ATCTTTAGCGATGGATAGACGAA

>LvLYZ1 JN039375.1

GCAGACACAGCCAAGCAACTTACACTTCGGAACCAGAAGACATAATGCGCGCATCACCATTC
TTCCTGGCGGTTGCCCTCAGCGCCGCGGTTGCGGAATTAGAAGACAGTTGCCTAGCCTGCAT
GTGTTACGTATCCAGTGATGGTTGCGTCATGCCAGATGAAGTGTGTAGAACCACATCATGGTC
AGAAGTGTGTGGACCCTGGGCCGTTACCAAACCTTACTGGGAAGACGCACATAAACCAGGA
GGAGAATTTTACACTTGCATGGGTGACTGGGACTGCAACGAGCAGACCGTCCGGGCTTACCT
GGACAGGTACGTAAGTAATCCTTACGCCAGCTGCGAGACCTACGCCCGCACGCACTACGGAG
GGCCGTGGGGGATGAATGAGGACTACGCGACCGATTACTGGCTACAGGTCAAGGACTGCCTA
GACTACGGGCTTTTCACACCACCTCCAAGTGTAGAGTGAAATACCTCGTGTTTATAGCCCAAG
AAAGCTAAGCTGCCGAATGTGGTCGCAGCAAAGACTGGAAAAATAAATCTCCAAAACAAAAA
AAAAAAAAAAAAAAA

LvLYZ1-F: TACGCGACCGATTACTGGCTAC

LvLYZ1-R: AGTCTTTGCTGCGACCACATTC

Supplementary Fig. 5

5' flanking region sequences and dual luciferase reporter vectors primers of 21 WSSV immediate-early (IE) genes. GenBank accession numbers are showed and the location of primers are underlined. The translation initiation codon of each WSSV IE gene is showed in red. The predicted LvDorsal binding domains were shaded in grey. The unique p53 response elements of wsv403 promoter were shaded in purple.

>wsv051 AAL33055.1
TCTTGGAAAGAGGAGTGGTGCCCAGAGATGGGAGTAACACGTGGCTCCACACGGACC
TTGTGCAACTCTCCACCTCCATATTTAGAAGTATTCGCAACAGAGGAGTGAATATTG
GCGGTAACAACAACACTGGTAGCAATTCTTCTAGTTCTTCTTGTGGAGGGAATAAGG
GCGATTATGGAGTACGTTGTGGATTGAGTATAAGCAAGCGTGGTATAACCCTGAAAC
CACCACCTGCAGCGATGACTAATTCTTCTTCCCCGTCATCCTCGGCCATGATCTCAT
TGCCTCAGCCCACGCGCCAGAGCATAGATCTTTCGATAACGACAATCATCCAAGATT
TCTCAGAAGTTTCTGGGAAATTGAGGCTTAATGGATTACAGAAAAACATGTCTGACA
AGAGCAAAGACGTGTTTAATGATGCAATATACGACTCTGGCGCATTCAAGGCGCTCC
TAACATGCACAGTCAACGATAAAAGTAGACGTAAAAGGAAAAGAAGGACTTTATTGG
CATCTGGAGAGGGTGTGGTACGAAGAAACCTGATGGTGAGTCAGGGCAATGACGTCA
ATGATGCCCACCAGTTCCAGGAAGAATGCGGAATAAAAATTGGGGGCGGGGCTTCTA
GGGTGTATAAAAGAGCCCAGCGCCGAGGTTCGGCAGTCAGTTCCAGAAGAAGAGTAA
GGAACAAACCCCAGTTTACTATAGCAGTCTCTGACGAAGACGACGACTGCGAAGAAG
AAGGCGACTTTTCTTCCGAGTTAAATCCAACGCACTCTCAACTACTTCTATTCCAAC
AACGGCAACAAGATAGCTGCACAGAAGACGACGACGTTTTAGTGTCTGTAGAAGAAT
ATAACAACAGAGTAAGCGGTTCTTCCACCACAGCCGGAGACAGAGTTCTTGCAAAGG
ATCTTCTCTCTACTGTATCTCCGAACGAAAAGAGGAACTCTGCCGCCCTCGCCGCAC
TCACCATATCCCGGCACTCTCTTTTCAACGCTCTATCTGCAAAAACAAAGTTGGGAG
AAAATGGACGTTTCTTCCTATAAGAGCACTATTGACTACCACAACATTGAAGATATG
GACGATCTCCAGCGCGCCA
wsv051-F: GGGGTACCTCTTGGAAAGAGGAGTGGTGC
wsv051-R: GGCTCGAGGAGATCGTCCATATCTTCAATGTTG

>wsv056 AAL33060.1
CTATCTCTAAAATCAATCTGCCGTAAAGGGTCGTCATGTTCATCCCTTTCTTCTTCC
TTCTTCTCAGTCTTGATAACTGGAGCAAATTTCTGGAAGGAGTTTGGGGCCATTGTT
AGGCTATGCGGAGCAACACAAGAGTGTATTCGGCAGTGATGGTGGATGAAATGTGTG
CAAAATATGCTTAGTGGTTAGCTTATATAGTCTGCGTCAGTGGGAGAAAAAATGTCC
CAGAAACTAGTTGGGATGTTTCTGGGCTCTAGCCACTCTAGAGATGTCTGGAGCGCA
CCCTAGCACCCTCTTTTTCCCCCTTCATCTTCATCTCAAAAACTTTTCAAAAATTTT
TCTGGGTCACTCCAGTTTAGGGGGTGGACCGCTGAGTCGATCGAATGTCAAGTTCCG
AGGGTGGACCGCTGGGTCGGCCCAATGTCAGATTACACGAAGGGGCTAGAAATGTAT
ACCAGAAATAATGCACAGAAATCTTTAGAATCATTTCTGGAGCAAGGCAGAACTGCC

TGTATTCACTAACCCTTTTCCCCCTTCATCTTCATCTCAAAAACTTTTCAAAAATTT
TTCTGGGTCACTCCAGTTTAGGGGATGGACCGCTGAGTCGATCGAATGTCAAGTTCG
AAGGGTGGACCGCTGGGTCGACCCAATGTCAGATTACACGAAGGGGCTAGAAATGTA
TAACAGAAATAATGTACCAGATGTGAGTCAAACCGTTTCTGGGTTCTAGCCGACCCC
GGTATGAAAGATGTGGGAGAATTTTTGGGGGGGGGGGTGTTAGTCTATATATAAGAC
TGTGAGCTCCTTGCTAGTAACCACACTACTACTTGCTCCTCTCTCAGCAGCAACAAC
CAAAAAAATGGCCTCAGTCTTTGAAGACCCTGCTGATCTCTTTGCCAACATGGACTT
GACTGGCAAAGTTCCTACCCGCCCCAATATCCTGTTCTTCGAAGGCCTACTCCCCAA
TTCTGGCAAGGAGATTATGGAGAA

wsv056-F: GGGGTACCCTATCTCTAAAATCAATCTGCCGTA

wsv056-R: GGCTCGAGTTCTCCATAATCTCCTTGCCAG

>wsv069 AAL33073.1

ATGTGGCTAATGGAGAATTGTCGTGCCAGTTGTATCAGCGAAGCGGAGATGTCGGGT
TGGGCGTGCCCTTCAATATTGCATCATACTCTCTTCTGACTCATCTGATGGCCAGTA
TGGTGGGTCTAAAACCGGGAGAGTTTATCCTCACTCTTGGTGACGCACACATTTATA
ATACCCACATTGAGGTGTTAAAGAAGCAGTTGTGCCGCGTCCCTAGACCATTCCCTA
AGTTGAGGATTTTAATGGCTCCAGAAAAAATTGAGGACTTTACTATCGACATGTTTT
ATCTTGAGGGGTATCAACCACACAGTGGAAACTTGCAGATGAAAATGGCTGTTTGAA
TCATGTTAAGGAATTTCCTTGTTACTCATTTATTCCTAGAAATGGTGTAATCGCTGT
TGTGGGCGGAGCATATTTGTGTATATAAGAGCCCGTGTTAGCTCCTCGATTCAGTCA
CAAGAGCGCACACACACGCTTATAACTAGCTCTCTCTCTCCACTCAAGATGGCCTTT
AATTTTGAAGACTCTACAAATCTCTTTGCCAATATGGACTTGACGGCTGGCACAACA
ACAGACCCTACCCGCCCCAATATCATATTC

wsv069-F: GGGGTACCATGTGGCTAATGGAGAATTG

wsv069-R: GGAGATCTCTTGAGTGGAGAGAGAGAGC

>wsv078 AAL33082.1

CTTCCTGACTTGACTGTTCCCCCTAGAAGTAACAATATTACCTTTGATACTATTAGT
GGTATTAGTAGTTCACTTTATGATGTTAATGATGATGATGATGATGATGACACAATG
TCTTTGCCTGACTTGAATATGCCTAGTGCTTCTACGTCATCCGCCCCTACCTCATCC
GCCCCTACCTCAACCTCTCTTAACATTAATGTTAACCTTTGTTTTAATGTTGATTCA
GACTCTGATGACGAAGAAGTAATACCTTCTTCGTCATCAGTGAATCAGCCCTCTACT
TCCTCAGGAAGTAGTAGTAGTAGTAGTAGTAACAGTAGAAAGAGGCCAAGGTATGGGCGT
GACGAGGACAGGATGTCTAATATTTCCTCTGAGAGTAAGAGACTGTGTGTAGATGTC
AAGAGGTATATGTGTAGACTCGATAATATTGATGAGGAGTATAATGAGATTGCCAAT
AGGTATCTGGCTGAACTTTCTGCTCTTAGAGAAAGGAGACAGGAAACTGAGAATAAG
CTTGGAGATTGTATATCTAGAGGTAACCTGTTCCATACAACTGTCAATGATGTAATT
GGTAAGAGTTTGTGCTCTAAGAAATTGAAGGTGAAGCGTAAGTACGCTTCAAAGTGG
AGTGCTAATAAGCAGCTAATTGGTTCCTGTCTCATTAAGTCAGCCTCTAATAATGCT
AGGTTGGATGACGAAATTGCACATGTACACAGTTCATTGTTGAATGGGTTTGATACT
GACCCTTCGGAAGCTGATCAAATATCTTCCCTGCCCAATCTATAAAGTGATGTGTTT
TTGTATATGCCTGCCTGTAAATGATGTACAAATTTTCTACTGCCTTTTATTAAAATA
AAAGAATGTTACATTATAATGCATTTTATTACATGTGTTACCCCCTTGTTTTTTAGA

GTCATTAAAAATGGCCGGAACTGACATCATCAGCAGCAGCAGCAGCGGCAGCAGCAG
CAGCAAGAAGGGAGGGTGTATCGTCAGCAAGAAAGGAAAGACAATAAAAG
wsv078-F: GGGGTACCCTTCCTGACTTGACTGTTCCCCCT
wsv078-R: GGCTCGAGCTGACGATACACCCTCCCTTC

>wsv079 AAL33083.1
CTGCTCGTGGTTGTTTGTCTGATTTGGTCGAAGTGCCTGAAGATGTGAAGGACTTTA
ACACTTTCATCTGTCCCTGGGAGACCTTTTTTGAGATTAAATATGGGGTCTATTACA
TTGTGAATAGGGGGACTGTTGTCAAGTTTATGAAGGATATGAACTATGAAGAGTTTG
TTTTTGAGTGTGTTAATGGCCTTTCTGTATACAGAAAGAATATTAAGGGGGTAGTTG
GGGTGACTGGTGTGTGTCCTCAGGGGTTATGTTTAGAGATGCCATTTGCAGGTATCA
GTATTGATGATGTCATTAGGTGTGTCAAGGATAGTTTAGATGGTGGGGAGTATTATG
AGTCAAGGGACGCACGCTTGTTGTATGGGGTTGTCATGCTTCAAAGGATGGGACGTT
TACCAGAGGTAAAGGGGGTTGATACAGTCGCACCAATAACAGACTCTTTCATTGCCC
GAAAGGTTGTAAGAAGTATGTTTGAAAAACTAAAGGTGAACATGCCTTTTGTTTTGG
CTGAGACTTGTAATGTAATTACAAGAGTTGCAAATGAGGGAATTATTAATGTCGATA
TAAAGGCTGATAACTTTGTTATAGATAGCATATCTGGCCAACCTAAAATGATTGACT
TGGGACTCTCATACCCTCTAGGTTATTGTTACAACGATGAATATTTTAGGAACACGG
AAGAACTAATCAGGCAGTACATTCACACACCTCCCGAGTTCTTTAGGGGACACTGTC
TAGGTGCCTATTCAATGACGTACAGTTTCAGTGTAATGGCTTCCAGTATACTGGAAG
ATGTTGTTGCTTGTTCTAACATGGAAGGCCCTGCCTTTAATTTGATGTCAAACATGC
ACTTTTTGATGTTGTTGCAAAGCGGAACAGACACTGATTTCTATCAAAATCGCCCTT
CAATCACAGAATATGCCCTTGCCATGAAGCACATATTCCCTTTTAAGGGGACTGTAA
TGAACCTGTTTAAAGTAAAGAAATGAGTTGCTATAGCGTTGGCTTTTCTCCCTCTTT
AAAGTAAACAACAACAACAACAAGATGGCTGAAACCGTCGCCGTTGATGAGGTG
wsv079-F: GGGGTACCCTGCTCGTGGTTGTTTGTCTGA
wsv079-R: GGCTCGAGTCAACGGCGACGGTTTCAGC

>wsv080 AAL33084.1
CCCTACAGCCAAGTCAGGCACAAAAACTGGCTTATTTTTTACATTCTTATAGGGAAG
AAGAGGGGGCAACACCTTGACAGGAGCAGGAGTGGGGGGTAAAGGAGTGGTATATTC
ACCCTTATATGAACTAGGTTTATGGAACTCCTGGCAAGGTATTTTGCCCTCCCTGTC
CTCTTTTAGAGGGGCGGCCTTACAGCCTCCAAAAACCTTCTTAGCTATTTTAGTATG
CATTTTCTCAATCCTTTTCATACCTTCAGACAACATCATGAACCCATTCTTATCAAA
CTGAAAACCCCTCAAGAAAAACTTATAAAAGGACTTTTCATTCTTTGCAAGAAGACA
TCCCGCCATTAGCCTAATTGCACTCAAAACATCCCCTATCTCCCCTTCCCCATACTT
TATTTCAGGAAACCTATTGAGTGCTGAGCAGGTACTTTCTATAGATGCTCTCAGAGC
ATCATTTTCCAAGGTTGATGAGCTATAATCAACCTTCTTACAGCTGGCACACTTCTT
AACCATATCCTTTATTTCCATTCTTTTCTTATGTTTCTTCATCATTTCTGATGAAGA
GGAAGTTTTGATTGATGTCTTAAAGACGATATTCTTCCCTTTTATTGTCTTTCCTTT
CTTGCTGACGATACACCCTCCCTTCTTGCTGCTGCTGCTGCCGCTGCTGCTGCTGCT
GATGATGTCAGTTCCGGCCATTTTTAATGACTCTAAAAAACAAGGGGGTAACACATG
TAATAAAATGCATTATAATGTAACATTCTTTTATTTTAATAAAAGGCAGTAGAAAAT
TTGTACATCATTTACAGGCAGGCATATACAAAAACACATCACTTTATAGATTGGGCA

GGGAAGATATTTGATCAGCTTCCGAAGGGTCAGTATCAAACCCATTCAACAATGAAC
TGTGTACATGTGCAATTTCGTCATCCAACCTAGCATTATTAGAGGCTGACTTAATGA
GACAGGAACCAATTAGCTGCTTATTAGCACTCCACTTTGAAGCGTACTTACGCTTCA
CCTTCAATTTCTTAGAGC

wsv080-F: GGGGTACC CCCTACAGCCAAGTCAGGCAC

wsv080-R: GGCTCGAG AAGTGGAGTGCTAATAAGCAGCT

>wsv083 AAL33087.1

CTTCTGGCCTGGCCGCGCCGCCTTATATACACCGCTAGCGCCTGGGGGCATAAATGG
TGCCGGTTAGGGATAAAAGGGAAGACTGGGGTCCAGATCTGGTTATGGGTTGGCTCG
GCAAGAACAGGGTGTTGAAACTGGAGCGAGTGAATAAGGTGGGGGAATATAGAGGTG
ATGGGTGAGATGGGGGTATAGGTGAGATGGGGTGAGAGATATGGGGTGAGGTGGTGG
TAGACAGGGGACAAGGATAAGGATAGGATAAGAAGGACAAGGTGGGTCATCCAGATC
CAGATCTTCTTACCCAACGCTTACATGTTGTTATAACACCCTTAGGTCCTCCCCATT
TAGTGCCGGGGGTGGATTTAGACCCCTAAACAGGGTATGGCCAGGAGTGAACCCGTA
GCCAGATCTGGACCCCACTCTGTTGGAGAACTGGCGTTCGACGGCAAATTTCTGGAA
GTGGGGGTGAGGGGGGACAACTTGTATATAAGCGAGCCGGGGCAGGCCAGAAGCATC
AGTCTCAGCAGAGGCACAGCCAAGCATACAAGCTCCAGTTCCAGCTCCAGCTCTAGC
AGCCAACCCGAGCTTATCCAACGCTCTTACTACTACTACTACTACTACTAGTGCAGT
GCAGTGACAATAGTGGACTATTACCACTACTACTTCTACTACTACGTCACCAGTGCA
GTGTAGTGCCCAACAGTAAAACTGTTGTTTTCTGGATTTACTTCAGTCTCCGCTCCA
AACCCATCAAAAGTAAGTACCAATAATATACTATTATTGTTGTTGTTTTTTTTGATT
AACGTAACGTTAAATATTATGGGCTATTTTTTATATAAAAAACACATCACTTTATTA
GTGTGCCGGGCGTTGAATTAATCGTGTATTTGTTGTTGTTTCTTCCATAGGCAATGG
GGGGACCCACTGTAATTACTACTACCA

wsv083-F: GGGGTACCGCATAAATGGTGCCGGTTAGG

wsv083-R: GGCTCGAGTGGTAGTAGTAATTACAGTGGGTCC

>wsv091 AAL33095.1

GTAGTAGTAGTAGTAGTAAGAGCGTTGGATAAGCTCGGGTTGGCTGCTAGAGCTGGA
GCTGGAACTGGAGCTTGTATGCTTGGCTGTGCCTCTGCTGAGACTGATGCTTCTGGC
CTGCCCCGGCTCGCTTATATACAAGTTGTCCCCCCTCACCCCCACTTCCAGAAATTT
GCCGTCGAACGCCAGTTCTCCAACAGAGTGGGGTCCAGATCTGGCTACGGGTTCACT
CCTGGCCATACCCTGTTTAGGGGTCTAAATCCACCCCCGGCACTAAATGGGGAGGAC
CTAAGGGTGTTATAACAACATGTAAGCGTTGGGTAAGAAGATCTGGATCTGGATGAC
CCACCTTGTCCTTCTTATCCTATCCTTATCCTTGTCCCCTGTCTACCACCACCTCAC
CCCATATCTCTCACCCCATCTCACCTATACCCCCATCTCACCCATCACCTCTATATT
CCCCCACCTTATTCACTCGCTCCAGTTTCAACACCCTGTTCTTGCCGAGCCAACCCA
TAACCAGATCTGGACCCCAGTCTTCCCTTTTATCCCTAACCGGCACCATTTATGCCC
CCAGGCGCTAGCGGTGTATATAA**GGCGGC**GCGGCCAGGCCAGAAGCATCAGTTCTCT
GCAAGCCAGCAGAAGAGCAACACAACAAGCACTCTCTCTCCTTCTACCTAGAAGAGA
CCTGCCAATACTCAAGCTACAAGAATGGCCTCTCCAGCCCCCGCCGCACCAAGTCCT
TACACCATGTTGGACTCTAAGTTACTTAGTTCTGAGGAACTAAAGGAACTAACTTCA
TACGTCTCGACTAGCTCTCGCCGGTCTGATATGAAGAAACACTTGCTCCATCTATTC

GAGGAGCACGAGAAGATCTTCCAATTCATACAAGGTAAGCACAAGTTCTCACTATAC
ACTTTGGACTTTGAAATTTTCTATGTTATGCTGAATATTTTGTTGGTTGAAGTGAAA
AATATTCTAAGTCCAATTCCTTTACTCTTTGACAGAAATCTCCAACCAGTACGGAGA
CTATGGATGTTTCACAATGGCCCCGCCTCACCTGAACGCTGCAGCCGATCTCTT
wsv091-F: GGGGTACCCCCACTTCCAGAAATTTGCC
wsv091-R: GGCTCGAGGAGATCGGCTGCAGCGTTCA

>wsv094 AAL33098.1
AAACTTCTAAAACTAGGTGGAGACTGTACAACAGTCTTCTTGACATTTTGAGTATTG
ATTGAGCATTGTTGGTTCTGTTTCTTATCACTTGTGGAGGAAGAAGAGTGTTTACGA
GAGTGGCTTTTATTATGGCTTTTAGGCTGTGCTTCTGCATCACTATCTGATGACGAC
ATGCATGTTGTACTATTAACATTGTTGTATGAGTTATTTACATCACTATTTACAGCA
TCATATTTAGGGGAATGTTTAACATGAGCTTTTGGTGACTTGTGAACCTTTGATGTT
GATTTCATATCCCTTTCATCCTCTGATTCTTCATCACTACTCATACAATGAGAAGAA
GAAGCAGAACCAGAGGAACCACGGTGGGACTTGTTACGCCTCTTTTTCAAGTGCTTC
TTACGTTTAGGGGCTTCCTTTTCAACCTCATTTGTTTCACTATCATCATCAGTGTCA
AAAAGTGAACCTGCCCTAGACCTCTTAATAGAACTGCAATTATCAATATCCTTTCTG
GGGGCTGAGTTGCTAGAAGGGTGAATCGGGGAGGTAAAGCATGTGGTGGTTATAATG
TTATCAGTCTCGGTGGCTTTAAGAAGATCATCGAAACCTGTGCATTCATTATCAGTA
CCATCTGGTAGGGTGATGCCCTCAAAGAATTTTGCGGTCACGTCGCATTCGCCAGGT
TGCATTACTATATCGCCAGGTAAACCGTCCAACATGTCCAAAAAGTCATTTCCAACA
TCGGAAGTGCAAGTGGCAGCAGCGGGCACAGCTGCTGGTTCAGTAGGGGAAGTATTA
CCAGCTACTGAAAGGTCATCTCCTCTTACGAATGAAGAGTAACTAAATCCTGTCTTG
AGTGCAAGTGTCGCTGCCTTGGGTGAGGTTGTAGCAACGATAGGCTCAGGCGCAGGA
ATTTCTTCCATCATCTCATCAACTGCAGTCTGGAGTATGTCCAATGATGGTGATAGT
TGTTGTTGTTGTTCTTCTTCTACTCTCATTCTCTTGGGAGA
wsv094-F: GGGGTACCAAACTTCTAAAACTAGGTGGAGACTG
wsv094-R: GGCTCGAGACCATCATTGGACATACTCCAGAC

>wsv098 AAL33102.1
AATTGCAGTTCTATTAAGAGGTCTAGGGCAGGTTCACTTTTTGACACTGATGATGAT
AGTGAAACAAATGAGGTTGAAAAGGAAGCCCCTAAACGTAAGAAGCACTTGAAAAAG
AGGCGTAACAAGTCCCACCGTGGTTCCTCTGGTTCTGCTTCTTCTTCTCATTGTATG
AGTAGTGATGAAGAATCAGAGGATGAAAGGGATATGAAATCAACATCAAAGGTTCAC
AAGTCACCAAAAGCTCATGTTAAACATTCCCCTAAATATGATGCTGTAAATAGTGAT
GTAAATAACTCATACAACAATGTTAATAGTACAACATGCATGTCGTCATCAGATAGT
GATGCAGAAGCACAGCCTAAAAGCCATAATAAAAGCCACTCTCGTAAACACTCTTCT
TCCTCCACAAGTGATAAGAAACAGAACCAACAATGCTCAATCAATACTCAAAATGTC
AAGAAGACTGTTGTACAGTCTCCACCTAGTTTTAGAAGTTTTAGTCCTAAGAAAGAT
GAGCTTGGTGATTTCTTGTCACGCAAGCACACAAAGCCAGTTAGGCCCTATAACAAG
AAGCGTGATAATGTTAACACCACTAATAATGTAGTACAGAGGTCTGCCTGACCGACT
CAAATGATACTCAATCAATGTACAATAATAATCTTAGTACTTAACAAGAAGAACTAT
ATTTTTATAATATTTTTACATGTCTTAATAACAAACAAAATAAAAGAAAACCAATGT
ATTATATGTTTAAAATCAACCCCATTTGCATGATTAAACTAACTATAGTGTGTAAGG

AAAGAAAAAAACATGATTATTTCTGCCATTAAAACAACAACAAAAATTCTAAGCTT
CTCCTTTCTTTCTGTGTCTTTGCAGATTCGGGAGTCTTTCTAAAACCACAGCAATAC
AAAAAACAACACAAATTCTCTACTGCTCTCCCTCACCAATCCCCCTATCGCCCTTAT
GCTCCTTCTCCTCTCTATACTTCCTCCCCATTCACTTCTGACTATCAATTTTCTGAT
wsv098-F: GGGGTACCTTGCAGTTCTATTAAGAGGTCTAGG
wsv098-R: GGCTCGAG AAAATTGATAGTCAGAAGTGAATGG

>wsv099 AAL33103.1
ACACCCACTGATGAAGTCTCTATGGGGTTGGTATTTGTTCCCATTGATTGGAATTCT
TGCATATCAGTATTTGTTCCCATCGTTTGGAATTCTTGCACATCAGTGCTTGTTCCC
ATATTACTAGTGTCAGGCGTGAGACGCCTCTTCTTACTGGCACTCCCATTGGTCTTT
TGTTCTTCCCTCTTGTGGAACACTACAGTATCAAAATTTTCTCCTGTTGCATACTCT
GACGCATCGCCATGTATTCCTCCATCATCTTTATCAAAGCATACGACAACTCCATTA
ACTATCCAACAAAGGTTAGTATAGAATGTCATATCACTGCACAGTGTGTCTGCACGA
GCAATCAGATTGGGGAAAGCTTTGTTCAATACTCTTGAAACAAACATCCTTGGGACC
TTGTATTCATCTCCATGTTCAGCCAAGAATCTTTGTTCCAAGTGAGCCAAAAATACA
CCTCCCCTTTTACCGGGAGGGGCCATGCATACATTCTCGTGAATCCATTTCCTCAAA
GCCTTTCTCCCCTTAAGGAATTGACATTTCTTGGACCCTTTCTTATTATGCATGGCA
GGTGTGGGAGGAGGAAATACATAGGAAGAAGTATACACATCTATGGATGGGGTGGGC
AATTCAGCGCAAAATTGAGGAGGGGCGACACCGCACATATCGATACTAGGGTCAACT
TTATAAAGGGAAATGTTTGCCCTTGTTTTATAATCTGCTGCGACAGGAGGTTCTCCT
CCTACATATAGGTGAGGGAGAGGATCTTTCATCATCTTTTTGAGTTTCTTAATTTCC
TTCCTCTTTTTCTTTTCCATTTTTTTCAATTCAGCTTCAGAGATCCCGACAGGGATA
GAAGAAGAGGCGGCAATAGACGGTGCTGATGCTGCTGCTGCTCCTTCTTCGTCACAT
TCATCCGAATCAGTATCCATGATGGATCGAGGAGTAGGAAGTGGTGTTGTTGTAGAG
GGAGGAGGGAAAG
wsv099-F: GGGGTACC ACACCCACTGATGAAGTCTCTATG
wsv099-R: GGCTCGAG CCCTCTACAACAACACCACTTCC

>wsv100 AIX03665.1
CACCGTGGTTCCTCTGGTTCTGCTTCTTCTTCTCATTGTATGAGTAGTGATGAAGAA
TCAGAGGATGAAAGGGATATGAAATCAACATCAAAGGTTCACAAGTCACCAAAAGCT
CATGTTAAACATTCCCCTAAATATGATGCTGTAAATAGTGATGTAAATAACTCATAC
AACAATGTTAATAGTACAACATGCATGTCGTCATCAGATAGTGATGCAGAAGCACAG
CCTAAAAGCCATAATAAAAGCCACTCTCGTAAACACTCTTCTTCCTCCACAAGTGAT
AAGAAACAGAACCAACAATGCTCAATCAATACTCAAAATGTCAAGAAGACTGTTGTA
CAGTCTCCACCTAGTTTTAGAAGTTTTAGTCCTAAGAAAGATGAGCTTGGTGATTTC
TTGTCACGCAAGCACACAAAGCCAGTTAGGCCCTATAACAAGAAGCGTGATAATGTT
AACACCACTAATAATGTAGTACAGAGGTCTGCCTGACCGACTCAAATGATACTCAAT
CAATGTACAATAATAATCTTAGTACTTAACAAGAAGAACTATATTTTTATAATATTT
TTACATGTCTTAATAACAAACAAAATAAAAGAAAACCAATGTATTATATGTTTAAAA
TCAACCCCATTTGCATGATTAAACTAACTATAGTGTGTAAGGAAAGAAAAAAAACAT
GATTATTTCTGCCATTAAAACAACAACAAAAATTCTAAGCTTCTCCTTTCTTTCTGT
GTCTTTGCAGATTCGGGAGTCTTTCTAAAACCACAGCAATACAAAAAACAACACAAA

TTCTCTACTGCTCTCCCTCACCAATCCCCCTATCGCCCTTATGCTCCTTCTCCTCTC
TATACTTCCTCCCCATTCACTTCTGACTATCAATTTTCTGATTCATTTCAACAACAA
TGCCACCAATACAATTACTACCCCACACCCCCTCCATCTATGTCCTCGACGACCTCT
CCATCGTCTTTGTGGGATGATGACGATGATGATGACGAAGAAGACGAAAAAGAT

wsv100-F: GGGGTACCCACCGTGGTTCCTCTGGTTCT

wsv100-R: GGCTCGAGGATGGAGAGGTCGTCGAGGAC

>wsv101 AIX03666.1

TACAATATTGTCCATGTCTAATTACAGTCTTCGCCCATTTGCACATGGCAGTACATA
CATCCATATTACAAAAGAAAGCGTGGGAAATGTTACTACAGAATTTGGGGACTGTTC
CTCTACGCTTTGCAAGAGAGAAGGATCCCTTGAACCACTCCTGGACAACATTACAAG
TCTGTCCCTTGTTATATGATGGAACAAGAGGGGATAGATATTTGGCCATATACTCAT
AAAATGGATGAAAGACTTTATCCTCTTCGTAATTGTAAGAATTGGGAATACTTGGAT
CATCTTCGTCACATTCTAAGGGGAACGTGGGAGGGACAAATCCATTAGGCACTACTA
CATTCGCACCTCCTGTGAAGCAGGTAGGAATTGGGGTGTTAGACAGGGGAGATGTTC
CTGTATTTGCACTCATTCTAGAATTTCCACCTCCTCTGGAAGGCGAATAAAGCCACA
TCATGTCCAATTCTGGTACATCATTCGTTAAAGGAGTAATTACCAATCTTGGGGGAG
GGTTGGGAAGTGGGTTGGTATTCACACCCACTGATGAAGTCTCTATGGGGTTGGTAT
TTGTTCCCATTGATTGGAATTCTTGCATATCAGTATTTGTTCCCATCGTTTGGAATT
CTTGCACATCAGTGCTTGTTCCCATATTACTAGTGTCAGGCGTGAGACGCCTCTTCT
TACTGGCACTCCCATTGGTCTTTTGTTCTTCCCTCTTGTGGAACACTACAGTATCAA
AATTTTCTCCTGTTGCATACTCTGACGCATCGCCATGTATTCCTCCATCATCTTTAT
CAAAGCATACGACAACTCCATTAACTATCCAACAAAGGTTAGTATAGAATGTCATAT
CACTGCACAGTGTGTCTGCACGAGCAATCAGATTGGGGAAAGCTTTGTTCAATACTC
TTGAAACAAACATCCTTGGGACCTTGTATTCATCTCCATGTTCAGCCAAGAATCTTT
GTTCCAAGTGAGCCAAAAATACACCTCCCCTTTTACCG

wsv101-F: GGGGTACCTCTAATTACAGTCTTCGCCCATTTG

wsv101-R: GGCTCGAGAGATTCTTGGCTGAACATGGAGA

>wsv103 AAL33107.1

CTAGGAAGTGGTGTTTTTACTTGCGTGTGATTAACATTGAGGGATGAACCCCTAGCC
AGATTCACACCCCTCTCTAAGACTCCCCTGTGGTCTGCTTTAGGTCAGGGGTCTGGT
CTGATTCTGCCAGAGTGTAGTTTCAAAAATACATCATTTTATATGTATATTTTTAAA
GGTTTTTAAAAAAATAGAATCATATATAAAACATTAGTTTATTGGTATCTTATTTAC
ACAATATACAGAAAATGTCACATATCAACCAATCAATCAGTCATCCATGTCGTTGCG
AACCCTTTCCCTGCACTTGGGAACATCACATGAAGCGTCTCTGCAAACAATGTAGTG
ATATGCGAGCATGGATGTACATGACCTCCTTACGATACATCTATTACAATATTGTCC
ATGTCTAATTACAGTCTTCGCCCATTTGCACATGGCAGTACATACATCCATATTACA
AAAGAAAGCGTGGGAAATGTTACTACAGAATTTGGGGACTGTTCCTCTACGCTTTGC
AAGAGAGAAGGATCCCTTGAACCACTCCTGGACAACATTACAAGTCTGTCCCTTGTT
ATATGATGGAACAAGAGGGGATAGATATTTGGCCATATACTCATAAAATGGATGAAA
GACTTTATCCTCTTCGTAATTGTAAGAATTGGGAATACTTGGATCATCTTCGTCACA
TTCTAAGGGGAACGTGGGAGGGACAAATCCATTAGGCACTACTACATTCGCACCTCC
TGTGAAGCAGGTAGGAATTGGGGTGTTAGACAGGGGAGATGTTCCTGTATTTGCACT

CATTCTAGAATTTCCACCTCCTCTGGAAGGCGAATAAAGCCACATCATGTCCAATTC
TGGTACATCATTCGTTAAAGGAGTAATTACCAATCTTGGGGGAGGGTTGGGAAGTGG
GTTGGTATTC

wsv103-F: GGGGTACC CTAGGAAGTGGTGTTTTACTTGCG

wsv103-R: GGCTCGAG TGGACATGATGTGGCTTTATTCG

>wsv108 AAL33112.1

GGGGAGATCGTAGAAATGGAAGGTTTTATTGACATTCCTTTTCTTGAAGGTTTCGAA
AATATCCTCGCAGAACAAAGCAACGAAACTGGTGTGACATACCCTAATACGAATCAA
GATGTGGAAGAAAAAGATACTAAAAATATAGATGTCGTCAGAGAATTGGAAGCTGAA
TTTAGTAGTGGAATTGGGAGTGGCTCCATGGACTCTTCTGACTCATCCGATTCTTCT
TCTTCTTCCTCTGACTCATCCGATTCGTCTGATTCATCTGACTCTGAATCATCTGAT
GATTCAGAAGGAGGGGATAATAAGGTCCGAAGAATAAGACGTCATCAGTATCACCGG
CGCCAGTTGAGTTATTCGGATGACGTCAATGGAGGGGGAAGAAATTCTGAGAAAATG
GAGATGGACAGAGTAACTCACATAAAAACTGAACACATAAAAAGAGAGGACGAACCC
AGATACGAAGAAAGAGAAAGATATATTCATCCAAGAAGAATGCAAGTGCCCAAGGAC
TATTATTGTGAGCAATACGAACACTACGACGCCCCTGCTGCTGCTCACCACCACCGC
CACCACCAACACCGCCACCAACACCAGAGGCACTTTAACCAACCCCGCTCCAACAAT
TCTTCTGACGTTACTGCTTACGTCAATGAAAATTCCCCCACGAGGCCATGCCGTGAT
CGCAACTCTCGATTCTCAGAAAGACCCAACAATGGCGGTTATAACCGGATCAACTCA
AGGTATACAACTTTCGACCCTTATAGATATGGCGCAAGAAGAGGGCGTGGAGGAGTA
TATTAGTATCTGGCAACCCCCGAAAATCGGATATGAAAGGGGCTGTGGATCTGGGTA
GAGCACAGTCGACTACAAGTCCTCTCTGAGAACACAATAGTCCACAACCAACCAACC
AACCAAGAAATCAAGATGTCTCACATCAACTCTACCTCTGCTGCCACGACTTCATCC
AACACTCTGCCGATTTGCACCACTACAGCCC

wsv108-F: GGGGTACC GGGGAGATCGTAGAAATGGAAG

wsv108-R: GGCTCGAG TGGTGCAAATCGGCAGAGTGT

>wsv178 AAL33182.1

GGATTGACACTGTAGTTGTTCGAACGGTTTTCCTGTATAACATTTCCCAGCTGCGTG
GTAGTGACGCACAAAACTCATGCAAGAATGTGCGTGGGTAGTTTCATTTCTAAGAAT
CTCCCACGCACTAGAGGCACAGAAAAAATCAACTGCTGCGTGGCAGTGACGCACGGC
CACACACCCGCATTCTTCACACCATAAAAGGACATGATTCGTGTTAGTCGTCACATC
TCTCAGAAACCCCCATGTGCATTGACGTCATGGGGAATTGCTATTGCACCACCTTAT
GGTGTATAAAAGTGGCCCTGGGTGGCATCGGTTAGTAGACAGAAACAAACCGTCAAG
ATGGTGTCGTCTATTACCCACCTCTCTCTGTT

wsv178-F: GGGGTACCGGATTGACACTGTAGTTGTTCGAAC

wsv178-R: GGCTCGAGAACAGAGAGAGGTGGGTAATAGACG

>wsv187 AAL33191.1

ATATTGTCCATGAATGGGAAGGGGTTGGTAACATTATAGTGCTTTTCTAGTCCCATT
TCAACCAATAGTTTATCTGCCACAAATTCAATGTACTGGCTCATCAACTTGCAGTTC
ATTCCCACTAATTTAACAGGCAGGGAAACTGTGAGAAATTCTTGTTCAATTCGGACG
GCTTCAGTGACAATTTCAAGAATCCTTTCTCTTGATGGGGTATCAACAAAACCCTTT

TTCAACAGCATGCATGCAAAGTCGCGATGAAGACCTTCGTCCCTAGAAATGAACTCA
TTGGAGGAGGTGAGACCAGGCAAAATACCCCTGTTCTTGATCCAGAAAATGGATGCG
AATGCACCACTAAAGAAGATTCCTTCAACTGCAGCAAAGGCAACAATTAGTTCCGCC
AAATCGTTATTGCTTTGCATCCAATTAATAGCCCACTGCTCCTTCTTCTTGATGGCG
GGGAAGTGTTGTGCAGCGTTAAACAAGATAGCCTTGTCTTTTTCGTCGGGCACCAGT
CTATCAATCAGTTCTCCGTAGACGTTGCCATGAATACTCTCCATTCCAACTTGGAAG
TCAAAGAAACTCCTCGCTTCTGGAATCTGCGCCACTTGACGAAGACGTGTTGTAAGA
TTTTCAATTACAATTCCGTCAGAGGATGCAAAGAACGCCAGAATCTGGAGAATGAAA
TCCTTCTCATCTTGAGTGAGTTTCTCCCAATCTTTAGGATCCCTTTCGAAATCAATC
TCTTCCAATGTCCAAAAACAGGCAACCTCTTTTTTGTACATTTCCCAGAGATCTTCA
TGGACAATGGGGCGGGATACAAAACGGTTGGGGTTTTCTCTTGTAAGTAAGTGTTTA
GACGTATCTACTTGCATAATGAGTTTTTTGGTGAATTTGTCAAACA

wsv187-F: GGGGTACC TGGGAAGGGGTTGGTAACATTATAG

wsv187-R: GGCTCGAG ATTATGCAAGTAGATACGTCTAAACAC

>wsv249 AAL33252.1

CTCGCCCACCACCAGATATCTGGGAAGCCTTAGGCGAGTCATGTTTCTTGCACCATG
TATTTCTAGGAATTTCGCTGACGTCAATAAACTGGTTTGTCATCCTGTTTTATCCAG
TTTGCTGACGTCAATAGACCATATTTGGGCATCTCGCCCACACAGAAACAGGATATG
ATATCATAGATTTCTGGGAAGAGGGTGTTTTTTGTGCCGATCTGGGTGTATAAAAGA
GCGCTGCGGAGAGGCAGAAACATCAGACAGACTTGATCTGTACAGCTAGCAGCAGCA
GTAGCAGCAGCCAAGAGAAGATCGGACGCAAACCATTCTCGCAGCCATGGCAGCAGC
AGCAGTCTCAGGAGAGGGGAGAATCTCTGCAGATCTACTCCTGTTGGA

wsv249-F: GAGAGCTCCTCGCCCACCACCAGATA

wsv249-R: CGCTCGAGGGCTGCGAGAATGGTTTG

>wsv358 AAL33360.1

AATTTTCGTATGCTGCAACTCCCATTCTGCAGACAGATTGCTTTTCAAGCACACTTT
TGGGTACATATATGCTAAGTTCTCGGTTAATTGCGTCGCACATGTGTCTAAACTTCT
CAAAACCTCCTTCAAATTCAAAATCTTCACGAATTATTTGCTGATAGTACTCTTCTG
GATGAGAAGTGATGTTGTACAGCGCACAAAACCGCTCAGCAAGAGTAACGACTGTCC
ACGCGAATATGGTGTCGTTCTTGTCAGCACCCGTGGCGAATGTTGACGTGATGATGG
ACAAGAGCTTCTTGATATTTTCCAAAGAAGCTATCTGGTTTTCATCAGATGACGAAG
TGGATGTTGCACCTTCTTCTAGACTAGAAACCATATCTGGTGTTGTTGATGATGTTG
ATGCAGCAGCAGCAGCAGCAGCTTCTTCTTCACTTTGAGTAGGTGGAGGAGGAATAC
TCTCCAATTCTTCAATTGTAGGAGGTTTTAGTGATTCTTTGTGGGTAAATTGCCACA
TTTCTGCAAATTTGCCCTTTGATAGTGCATCCCATGTGTTATTCTTGAGTCTTTGTT
GTAGAATGTCCGTATCTCTGGGCAATTTCAGGTTTCTGTTCTCATATCTCACAACAA
TTTGAAGGATGGCGAAAGGCATGATTGTGTCAAACTTGATGGTGTTAACGCCAGTCA
AATTCCTGATAATGTTCTTTGTTTCTGGTTTCACGCGTTCAACACAATTCTTCTTGC
TCATGGCTTTCTGGGGAAGAAGAAACTCCATTGCCAGAAGAGGAGAGTTGTCAATGT
CACTCAGAAAGAAACCGTGTTTTCTCTTGAAGAAACACACAATCTGGTCAAGCTTAT
CATCGTCATTGATGATGCTATTATAGACATTGGCAGCAAGGGGAGGGATTTGTTTAG
GGGCTGGAGGAGGCTTGTTGATGTTGTTCTCGTCTCCAGAACCGCCGCCAGATTTCC

CTCCAGATCCTCCTTCCTCATTATCTTCTTCCTCGTCGTCTCCATCTCCAGAATCGT
CCAATCCTTTACACGCCATCTTCTCCTTTAATAGGGTAGCATTTGTCTTGGTC
wsv358-F: GGGGTACC AATTTCGTATGCTGCAACTCC
wsv358-R: GGCTCGAGACGAGAACAACATCAACAAGCCT

>wsv403 AAL33416.2
AAAAATTTTTGATGAGGGAATATAGTACTCCGTAGCCAACATATACACATGAACACA
TGAGGCGGTCTACAACAGAAAGAGAACTGATAGCTGTTCCAGATATCTGGGTCGGCC
AGAACCCAGAAACGTTTCAACTCATTTCTGGACAAGCCATTTCTGGAAAGGGGTACA
ATTTCTTATAACTGGTATATCATTTCTGGTATAATTTCTGGCACCTTCGTGCAATCT
GACATTGGGTCGACCCAGCGATCCACCCTCCGAACTTGACATCGGGCCTACCTAGCG
GTCCACCCTCTAAACTCGAGTGAGCTGAAAAAATTTTTGAAAAATTTTTGATGAAGG
AATATAGTAGTATAGTGCCCGCCAAAGCATACACACACTCGCCCATGCTCGTCTAGC
TGATAGCTGTTCCAGATATCTGGGTCGGCCAGAACCCAGAAACGTTTCAACTCATTT
CTGGACAAGTCATTTCTGGAAAGGGGGTACAATTTCTTATAACTGGTATATTATTTC
TGGTATAATTTCAGGCACCTTCGTGCAATCTGACATTGGGTCGACCCAGCGGTCCAC
CCTCCGAACTTGACATCGGGCCTACCCAGCGGTCCACCCTCTAAACTCGAGTGACGC
AGAAAAATTTTTGAAAAATTTTTGATGAAGAGATTGAGTAAAATTTCTTGACGATAA
GAGGAGGCAGTAGGTGAGGCTGCTTGTTTGATGTGTCAGCCACATCTGCGTCATACA
TTATATTTCCAAGAATTTTGCTGACGTCAATGGACCATAAAAGGCTTTGTACGTCCA
GAGACAAGTTTTAGTCTGATAGATTTCTTAAAAAAAAGAGGGTGGGAGGTTTGTTTT
TGTGGGTTCTGTGTGTGTATAAAAGATAGGTGCAAAGGTAGAGAATCATCATATGGA
CAAAATCTGTCCATGAGACCTCAGTAGAGAACGCACCATGGTTGCTTCAACTCCGTG
TCCAGGCCCAGGACCAGTTCCAACCCAAGAACTTCTTTCTACAAACTTTCTTGAAGC
TCACAAGCTTGTCGTGGAACTTCTTCTCCCGTCCTACAGTAGTGATGTAGTTTATTG
TGACTCTGAGACGTACACCAAACCTATACCGATTTTTGG
wsv403-F: GGGGTACC AGTACTCCGTAGCCAACATATACAC
wsv403-R: GGAGATCT GTACGTCTCAGAGTCACAATAAACTAC

>wsv465 AIX03689.1
TCACCCTCATCCTCGATTCAAAAACTTTTGAAAAATTTTTTGAGCTCACTCCAGTTT
AGAGGGTGGACCGCTGGGTCGGCCTGATGTCAAGTTCGAAGGGTGGACCGCTGGGTC
GGCCCAATGTCAGATTGCACGAAGATACCAGAAATGTATGGTAAGTTATTGTACCAC
ATCTAAGAAATTGTAACCCTTTCCAGAAATGACTGTTCCAGAAATGGTTATAGACGT
TTCTGGAATCTGATTTCTGGACAGCCATTTCTGGAACAGCAGTGAACGGATGCTACC
GACCAGCAACCACTTTCTAACAGGTGTTTTCACCCTCATCCTCGTTTCAAAAACTTT
TCAAAAATTTTTTGAGCTCACTCCAGTTTAGGGGGTGGACCGCTGGGTCGGCCTGAT
GTCAAGTTCGAAGGGTGGACCGCTGGGTCGACCCAATGTCAGATTGCACAAAGGGGC
TAGAATATATGCTAGAAATAATGCACCAGTTATAAGAAATTGTAACCCTTTCCAGAA
ATGACTGTTCCAGAAATGGTTAGAAACGTTTCTGGAATCTGTCCGACCCAGATTTCT
GGACAGCCATTTCTGGAACAGGGCCGATCTGCCACCCTCTGAGTGTATAGTACGTAC
CCACTTTTCACCCTCTTCTTCATCTCAAAAACTTTTGAAAAATTTTTCAGCTCACTC
CAGTTTAGGGGGTGGACCGCTGGGTCGGCCTGATGTCAAGTTCGAAGGGTGGACCGC
TGGGTCGGGCCAATGTCAGATTGCGCCAGAAACGTATTGGCCAAAAAAACATCCTGT

CTTAGCCGGATATGACTAGTTTGAGTAATAGGAAATTGCACCATTATATAAACGTTT
TTGAAAAATGTCATCTCTAAAAATAGACCCTGTTTATACGTACGAGCGTCAATTGAC
GTCACACGAAAGGGGTATAAAAGCGGCCTGCGGAGGCACCTCAGCATACAGTTGTCG
AGATCGTTTCCTACCGTCAACATCAACTTCTACCATAAAGATATACTACAATGTTGG
GCCGTATCCGTTCAAGCGCTTCTTCCACCATGAGCAAGAGTTCTAGTTCTACTGTGA
AATCTGCATCTT

wsv465-F: GGGGTACC AATGTATGGTAAGTTATTGTACCACATC

wsv465-R: GGAGATCT TTCACAGTAGAACTAGAACTCTTGCTC

Supplementary Fig. 6

The genome sequences of LvFLp53 and LvΔNp53. The sequences of exons were underlined, the start codons of LvFLp53 and LvΔNp53 was indicated in red and in yellow, respectively. The potential poly (A) signal (aataaa) was shown in grey. All splice donor and acceptor sites conformed to the GT-AG rule and were shown in blue. The genomic DNA amplification primer sequences were shaded in green.

>Genome

AGTTGAGGAGAGAACAGTGATGTTGGTGGAAGTGTTGGATTTGTAAAGGCTAAAAAC
TCTGCCAAGAAACGCCCTCAGAAAGGATTTCAAAATAGTAACATCGACGCACACTCA
AGGGCGGG**ATG**CAGCGGTCGGACTCCGAGCTGCTGTTCGGCGAGGACGAGTATCACC
TGCTCAGGGATGACTCTCTCCTGCAACGCATCGGCTCCACCAACTTCCACACCCTGT
TGGAGACCTCAGACATTGTCGCCGTGCCCGAGCTGAAGGATGAGGAAGAACAGCATC
AGCAGCAACATCAAAATCAGCGGCAACAGCAACCTCAGCAGCAGCAGCAACAACAGC
AGCAGCAGCAGCCAGTCGCGCAACAAGTGGCTTACCCAATCCAGAATATTGATAACC
AG**GT**AATTAAAAAAAAAAGTATTTAGGGGTCTCTGGTAGAACTGTTAATTGCAATAT
GCTGAGGCGATATATAGGCGTACGCTTATTAATTGATTTTGACGTTGAAC**AG**CTGAT
CCTGGCGAATCCCCACATCCACGGAGATATCTTTCAAACTGTTCAAGTTCAGCAGCA
CACTTCACAGCAACAGCAGCTGCCACAGCTCCAGCCAGGACAGCAAATCCCGTGGGA
TTCTCTCCAGACTCTTGATACAGAAAATGTGGAAGTG**GT**ATGTATAGTCGCCGCTCC
TTTTCTCTCCTTTGCTCGCAGATGCAGATTCTCATTCATATTTCCACTCACTCACCC
ACTCACACTCAGACTCGCACTCATAGTCACACTCATAGTCACACTCATTCATATTCA
CTCACCGACCTAGACTCACTCACCCACCTAGACTCACTCAAACTCGCACTCTGAAGT
CACACTCACTCTTTCACTCCTTATTATCTCACTCACCCAAACTCAACTCAACTCAAA
CTCTTAGTCTCACTAACTCACTCACTCGCAGGGTTCTTACTCTTTATCTGCATTTAT
CATAAGTTCTGCCAAAGAAAAGTCTTGTCTGCATTATCATGGAATATTCGGGACGGC
AACGAACCATGACGTCATAGGCTTATGATGTCACAAGCAAATCATGACGTCACCGAA
GAGTTGGTCTGGTGATTCCACACCTCGGCAGTGTTTGGCACGACGGGTACAGAGTCG
CATATTTATTTTATAGAATTTAGTAATTGGAGGCGATTTCTTTTAATTGAGACTTAT
TTAGGGATTTAGGACATTGTGTTGAAGTTATTTGAAAGGAAATTCGCCTTTTGTTTT
CATTTTGATACATCGATATGCAATTAAGCGGACGGAGAGCACGAGCTTGCTCAAACT
TGTAGAGCTGGGCGGAGGTGCGGATGTACGATAACCTTACAATTTTCGCCGATATCT
TTGGCTCGTCTGCGCCCTTTCTTGGTGGTTTTCATCCCGTAATTAGATCCTGGACGA
CGTACTTCGACGTTCGGCAATCGCAAAGGATTACCGGAGTGACCCAGAAGTCCGAAA
AGGAGCAGAGGACCCAAAATGCGGGGGAGGAGGGGGGTGAGACGGGGTAGGTGAGGG
AGGGGGGGCTCACGC**AG**AGGGCAACTCCGTCGTGTCGTTACCTTCCTTCGGCACAGT
GAACGACCACACTACCTTTTCGGTGGTCGTACAGTTCCCCGAAGTCGTGTTTTTTAG
TCTTAAGTCTTTCCCCGAGACGAATCTTGTGTCGTCGCCACCGAAGCCAAG**ATG**ATC

ATCGTCGATAGG**GT**GAGTTAGCCAAGTCCCTGATGGCGATTTGTTTCCCTGGTCCAG
ATTTATTCGTCTTTGGGAGTCTGGTGAACAGTTGTTACCCTCTAATTGTCTAATTAA
GGTCGTTGGTACTTACGTGAAGCGGGTGGTTGGGTCGTTTTGAGAGCTGATCCCTGG
CGGACGCGAAATATGGTCTTTTTGAATTTATTTTCGGATAATTCGCTGGTCTTTCAC
GATTCTCTTGAGATCGCAAGATGGTTATGTTTTCGATTTTTCTTTCTTGGACTTGTA
ACCTCTTGAATTTGTGAAGTTCACGAAGAAGAGGTTTTGGTTGTAACCGTCGCGTGT
GGTGCTTTACGCAGGTCCTTGGATTGTATTGTCGTTAGTAATAGTCTTTTGTGATTG
TATGATATTGTTTAAAATGTGATAAAGGTAACGGTTCTTTTACACTCTTTGTATTTT
CGCGTTAAAGACGGTGCAAGGTTACGGGAGCGACAAGACGGCCGTATTATTTTGCCG
CGAAAATATCGGCGTTTCTTTTTGAACGTCAAATACCTCCGAATTTGCCTTTAAGAT
AAGGAAAAAAATAGTGTTTAAAAATTGGGGTTGAAAGTGTGCCCCATCGCTCCCTCGC
CCGCCCGCCCGCCCCTCTCCATGGCCCCCGTGGACAAAATGGTGGAGCGTCTTTGTA
GCTCTTTGCTCCAGGACTCGTCTTCGTCGGGTTCTTGGTTCTCGGTCGCAGTTTTAG
TTTTTGGAGGATTTCTATGGCGTTCGGTCCTTCTCTGTCTTCTCTCCTCTAATTTCT
CACTATGTCTTCCTTACCTCTTCTTGTCTCTCCTTTCCTCTCTTTTCCCTCTTCCTC
TCCTTTCTCTTTGTCTCTCATTACTTGCTCTATCCTTTCCTGTTCCTTTCCTCCTAT
CCTGCTCTTCCCACCCTTCCCTTCCCTTTTTTTCCTTTCTCTGCTTCTCTCATGTGT
ACGCTGTTTAGAATGTTGCCCTTAACAACCCTAAGTATAAATCAAGTTTGTATTATT
GTTTTAACAATGTATGTGGTACTAATTATATTTCCAGAAACTATGATTTCTGCAATA
TGGATACCATTAATTTCAGGCAAGAAATTTTTAAATCCTCTCATAGAAAGTTGGAAT
AGGTAACTAATAGATTTTAATGTGTTTTTATACCAAAAGGTATTTTGGTAATATATG
TGACAGACTAGGGAGAAACAATCATATAATAACCCTACATAGAGGCTAGTTATATAC
TGTATTTGTGTGCCCATCGCCTCATAATTTGAATACCCTTTGTTTACCGAGTCACCA
CGGTCTGTATGTCCCCTGTTTAGAATTTCCTACCAGGTACATACCAATTTGTTTACC
AGTTCTTATTCTTTACAATCCTCATTTCTGTTTATATATCATATTTTTTCCTCTTAT
TTCTTGTTCAAGCAGAGTCAGAGACTCATAATTATCATGATTACCATCGCCTTTCAG
TAGGATAAGTGTTAATTAATTTTTTTTCTCAAC**AG**TTGCCAAGCAATGATCATCGTC
GATAGGTTGCCATCGGTGGTGTATCAGCCGCCATTGCCTTGATTGAAGGCTCTACCA
CTTCCTGCCTGGCTGACTCTACTCTGGCACATTCAGTGCCATCACTCCAGCCATGGG
CAGGTCGTCACAAATTCGGCATCTCCCTCCCAACTGGCAACAAAGATCGCAACAAG**G**
TGTGCTGTGTTCAGAATTATCTTTTGTGGGGCCTTTATGAATGGTGCTTGTTCTTAT
TATTATCACTATGTAAGTGTAGTTCATTCCTGTGTATGGTACTGTCTGAACCTGTGC
TTTATTATTTTGTTTTTAAAGGAACTTTCTGAGAATAAGGACAGTTTATGGACTAAG
AATTAATTCTTTGGTTTTTATGCATAGGTATTTATATTATTTATAGTATGCATATTT
TTATTATGCAATATTACATGGTACAAATTAATATGAGACTGCCAATCCAAACTGAAA
CTAACATACTTTTGTTCTGC**AG**TGGTGCTACAGTCAGGATCTAGGCAAACTCTACCT
CTGCCCAAATGTTGCCGTACCAGTGAATGTAACATTGGATGACTGGGTGAATGCTAA
CATCACCATGACCCCAGTGTTCAAGCAAAGCTGCCACCGCACAGAACCAGTGAACAG
GTGCTACAACTGCAAGAGTATTCAAA**GT**AAGTGTGTAAATGTTCTTTTTAACACTTT

CCTTTCTTTTCTCCATTTATCTGTTGGTATTACCAGGGCAAGTTGTAAAGGCATTGA
AAATATTTCAGGTGTTTGCAAATGACTATTTCATGATTTCATGTCTTTTTAATTTTA
GATCGAAATAGAGAGGAATAATTTAAGATATATTAAGTAATTTTATTCTTTTCCCTC
TCCCCT**AG**ACTGTGATCCAAATTTGGCTGAGCATTTAGTGCAGGTCGAGGGTGAGGG
CTGTGAATACAGCTTCATCAACGACAGATACATGGTCACTGTGCCCCTCCGCCCCCC
ACCCCCTGGGGAGGTCTCCTCAACGCTCCTGATCAAGATTATGTGTCTGACTTCATG
CGTTGGAGGCCCCAACAGACGTCCCTTCTGTATTGTTCTTACTCTTAGAAACTC**GT**G
AGTATTGAGTTCTGTGTTATTAGATTGTGAAGAGTATGATGTTTCATGTTCTAATAA
ATTACCAATTATTGATGGCCTAATTTTAAATATGTTCTCTGAGTTTTTTGTGGTGTG
AAATATACAGTGGTTGAAATGGTTGTAATAGCTATAATTTTGCTCAACTCAGATCTT
GTAATAATGGAGTGGCTTGGGATACCTTGTGCTATAATTGATGACAAGCAACACCAG
AAGGTTAAAGAAATTGAGAACATCTGGTATTTTAACATCCTCTCTTTACATTTCATT
CCCTTTCATACTGTATTTATTTTTTATTATTAGGAAAGTAATGTATTTTCTCTGAAT
CGGGTGTAACCTTGATGTCACTTCAATATGAAGCAGATGGAGAAGGGATTTTCTTTT
CTGCTCTTTTTTGCTGTAATTTCTTAAAAAAAAAAGATGGTTTGGTTTGAATTCTGA
ATGTTCTTCCAC**AG**TGTTACTGGTGAAGAGATTGGTAGGCAGATCCTGGACATTAAG
TGCTGCAAGTGCCCATCTCGTGATCTGACTAATGATGAGAAAAGCAGGACTCCCACA
GCCCCTGCTGCACCATCAGCTGAAGAAGAAAAACGTACAAAG**GT**ATTGCTGCATTTC
ATGTTACTTTTTAATGATTACTGTGTGTCATATTTCATCTGAAATACATTATACATG
TATGAAAACTGTGTATGTATATGAAGTATTTTATATGATGCATGCTCACCTGAATAT
AGTATTAATGAATGTAAAAATGTATTCTGCAATCCCT**AG**GTACGAAAGTTGGCAACA
GAAATTGCCGTTGGCCAGAAGCGCAAGAGACCAAAGATCAAACTAGAACCAGGAACA
GATTCTCGAATGGTCAACATTGCT**GT**AAGTAAATTATATTTAACATTAGTTTGACCA
TAAATGAAAATTATGAAATGGTAAGAATTTTAGTCGATTAATAATGCAGACATAGGC
TCTTCTCCCCCATTCTTCATCCCCTTTTTTGCCTTTATACATGAACTTACACTGACT
TTGGGCTTAC**AG**GTCCCAATAGAGTATGAAGCTGAGGTGAAGTCTTACATCAACAAG
CTCATTGCCGCTGATTTAATCAAGAAGTGGCAGCCTGACGCGCTCATGTATCCTGAA
GAGGAGAGTAACTAAG**GT**AAACTAGACGTTGCTTACCTTAGGGCTGTCATGGTATAT
GACATGGACGTGATGTGTCCATCGTCACTGCTATCAACTCCATTTCAGTTCACTGTA
AAATACTAAAATAGAATTTTATTTGTGGAATCCAGATCAAATGTTCAATATTATACT
TAAATACATTGATTTACATGCCATTTAGGAGTTAATCTATTTTCAGGGGTTAATCTA
TTTTCCTGGCATTCTTTTTATTTTATTTTTTTATCAGTTTTAATTGTTAAATATATG
TCCATAATATTTATTTTATTTANTTTTCCTGGCATTCTTTTTATTTTATTTTTTTAAT
CAGTTTTAATTGTTAAATATATGTCCATTATAAATTTTATTTTTATTTATTTGCAAC
CTACCTTTCATGCCGAGTCGAACTTTAAGCTCATCTGTTTTTTCTTTTGTCTTTC**AG**
GTACAATTGATATTTCCTTTATTGATATTTTATCTATATGTAACTGGAACCTGTCAA
CACGGTGATCTGCGAGGAAGAATTGTGCACTAAAATAAATTTGCAATTGCTACTGGA
TGGAATTTTATATTAAGAATTTCACAATTGTTATTCTAGAAGTGTTGATAAGGTTTT
ACATATTGCATTTCTTGAAAGAGCTGTTTTTCTGTTAGGTAATAATCTTTTATATTT

AAGTTTTTAAATACCAGTGTTTATGACAGAAAGATTAGTTTAGGTAAGCATATTTCA
TTTTAAGCCATTGCCTATAACTTGTACTCAAGGGTATTTGTGTTTACTTGAAAAAGA
GAATGTCACTGTATCATTTTTGAGATTTTTTTCACAAAACATTCCGGTTTATTTGAA
AATTGTGATTAAAAACACATAGTATGTAAGTGCATGTAAAAGTGATGTTGAGTGTAA
TTCCAAAGCAAATATGTTGATTGGTGTTTTTGTATCTGAATGTTGCTGTTGTCTCAT
GTAAAAATGAAACTTTGCTTTGGAATACAGTGATTGTTTTATATTAGTCAAATAGAA
CATTTACTTCATTTGATTAGGTCAGAGAAGATATTATATAAAGTAGTTAGGTAGGTA
GGTACCAGCAAATACATAATATGTTTTGACTGTCAATTCTTTATTCTTTGCTATGAT
CAGTGTATGTATATTATTTTGAATAAAGGTTGACAAT**GT**TTTCTTTATGTACAGATT
TCCTTATATCTAGTCAGGTACGCATTCAATGTTTTTGGATCAGACATTTAGTTTTGA
ATACAGTGGTGTTTTACAAAGGAATACA
